# High seed diversity and availability increase rodent community stability under human disturbance and climate variation

**DOI:** 10.3389/fpls.2022.1068795

**Published:** 2022-11-30

**Authors:** Xifu Yang, Haifeng Gu, Qingjian Zhao, Yunlong Zhu, Yuwei Teng, Ying Li, Zhibin Zhang

**Affiliations:** ^1^ State Key Laboratory of Integrated Management of Pest Insects and Rodents in Agriculture, Institute of Zoology, Chinese Academy of Sciences, Beijing, China; ^2^ CAS Center for Excellence in Biotic Interactions, University of Chinese Academy of Sciences, Beijing, China

**Keywords:** climate variation, diversity-stability relationship, habitat loss or fragmentation, seed dispersal, seed-rodent network, species interactions

## Abstract

The relationship between diversity and stability is a focus in community ecology, but the relevant hypotheses have not been rigorously tested at trophic and network levels due to a lack of long-term data of species interactions. Here, by using seed tagging and infrared camera tracking methods, we qualified the seed-rodent interactions, and analyzed the associations of rodent community stability with species diversity, species abundance, and seed-rodent network complexity of 15 patches in a subtropical forest from 2013 to 2021. A total of 47,400 seeds were released, 1,467 rodents were marked, and 110 seed-rodent networks were reconstructed to estimate species richness, species abundance, and seed-rodent network metrics. We found, from younger to older stands, species richness and abundance (biomass) of seeds increased, while those of rodents decreased, leading to a seed-rodent network with higher nestedness, linkage density, and generality in older stands, but higher connectance in younger stands. With the increase of temperature and precipitation, seed abundance (biomass), rodent abundance, and the growth rate of rodent abundance increased significantly. We found rodent community stability (i.e., the inverse of rodent abundance variability) was significantly and positively associated with seed diversity, seed availability, linkage density and generality of seed-rodent networks, providing evidence of supporting the Bottom-Up Diversity-Stability Hypotheses and the Abundant Food Diversity-Stability Hypothesis. Our findings highlight the significant role of resource diversity and availability in promoting consumers’ community stability at trophic and network levels, and the necessity of protecting biodiversity for increasing ecosystem stability under human disturbance and climate variation.

## Introduction

Due to the accelerated impacts of global change, many ecosystems are facing a series of problems such as species extinction, biological invasion, pest outbreaks, and disease transmission (Patz et al., 2004; Pimm et al., 2014; Haddad et al., 2015), which is largely driven by the decrease in biodiversity and stability of ecosystems (Hooper et al., 2005; Dirzo et al., 2014). The relationship between diversity and stability is a focus of community ecology (Tilman and Downing, 1994; Rooney and Mccann, 2012), which has been debated in ecology for several decades, both theoretically and empirically (May, 1972; Thebault and Fontaine, 2010; Downing et al., 2020). Higher biodiversity can facilitate the temporal stability of aggregate community or ecosystem attributes (such as biomass and invasion resistance; Hooper et al., 2005), while high variability of local biomass could reduce ecosystem reliability (the probability that a system will provide a consistent level of performance over a given unit of time; Naeem and Li, 1997) and further increase risk of species extinction (Pimm et al., 2014). The relationship between diversity and stability at a single trophic level (e.g., in grassland ecosystems; Tilman and Downing, 1994; Downing et al., 2020; Yan et al., 2021) has been well studied. Recent studies suggest that horizontal diversity within a trophic level may increase the stability of food webs (Mccann et al., 1998; Zhao et al., 2019), while vertical diversity (number of trophic levels) might decrease the stability of food webs (Pimm and Lawton, 1977; Zhao et al., 2019). However, little is known about how resource diversity and abundance affect consumer community stability in complex forest ecosystems. Because food security is vital for the survival of consumers, we speculate that diversified and abundant resources would benefit the stability of consumers at various trophic levels.

This positive relationship between diversity and stability was challenged by May (1972) using multiple-species models, who found more species could not coexist together in complex systems with more links and more connectance. Subsequently, several hypotheses have been proposed to solve the diversity-stability paradox, mostly emphasizing the weak interaction at the network level between species, such as the weak interaction (Berlow, 1999; Neutel et al., 2002), nestedness (Bascompte and Jordano, 2007; Almeida-Neto et al., 2008), modularity (or compartmentation) (Olesen et al., 2007), and the diversity of interaction types (Allesina and Tang, 2012; Mougi and Kondoh, 2012). Specific density-dependent non-monotonic species interactions between mutualism, antagonism, or neutralism may facilitate the stability of networks (Yan and Zhang, 2014; Zhang et al., 2015). Variations in food resources (e.g., masting or non-masting years for seed-eating animals) could cause a transition of direct or indirect species interaction. For example, masting could result in the predator satiation effect (Jansen et al., 2004; Zhang et al., 2021a; Zwolak et al., 2022), a switch between mutualism and predation between seeds and rodents (Zeng et al., 2021), and promote mutualism via reciprocal seed pilferage (Vander Wall and Jenkins, 2003) or weaken competition between rodents (Vander Wall, 1990; Xiao et al., 2013). Masting could mediate apparent mutualism, predation, or competition between tree species through rodents (Yang et al., 2020). The impacts of network structure on community stability have been only investigated theoretically due to the difficulty of simultaneously quantifying species interaction and community stability in forest ecosystems using long-term data.

Habitat loss and fragmentation due to human activity is a major environmental issue of global concern (Haddad et al., 2015; Fahrig, 2017). In addition, climate change has a direct influence on ecosystem productivity and service functions (Garcia et al., 2014). Previous studies have shown that human disturbance or climate variation can alter species interactions (Rodriguez-Cabal et al., 2007; Richman et al., 2020), simplify the network architecture (Spiesman and Inouye, 2013), and threaten community stability (Mcwilliams et al., 2019). However, little is known about how human disturbance or climate variation can affect community stability *via* altering network structure.

In forest ecosystems, the seed-rodent interaction system is an important component of ecosystem function and services (Vander Wall, 1990; Yang et al., 2020; Hou et al., 2021; Chen et al., 2022). The population of rodents often oscillates greatly in forests under influence of climate change and human disturbance (Flowerdew et al., 2017). Seeds are important food resources for rodents, while rodents are also beneficial to tree species by facilitating seed dispersal and seedling regeneration (Vander Wall, 1990; Jansen et al., 2012; Wang, 2020). Therefore, the relationship between seeds and rodents can be either mutualism (in seed-rich years) or predation (in seed-poor years) (Zeng et al., 2021; Zhang et al., 2021b). Both the abundance and functional traits of rodents and seed species are key factors in the formation of seed-rodent interaction networks (Chang and Zhang, 2014; Yang et al., 2018; Liu et al., 2022). Although the succession stage facing human disturbance or climate change may have a significant impact on the species diversity and seed-rodent network structure (e.g., Yang et al., 2018), the relationship between community stability and seed-rodent network complexity has not been investigated.

**Figure 1:**
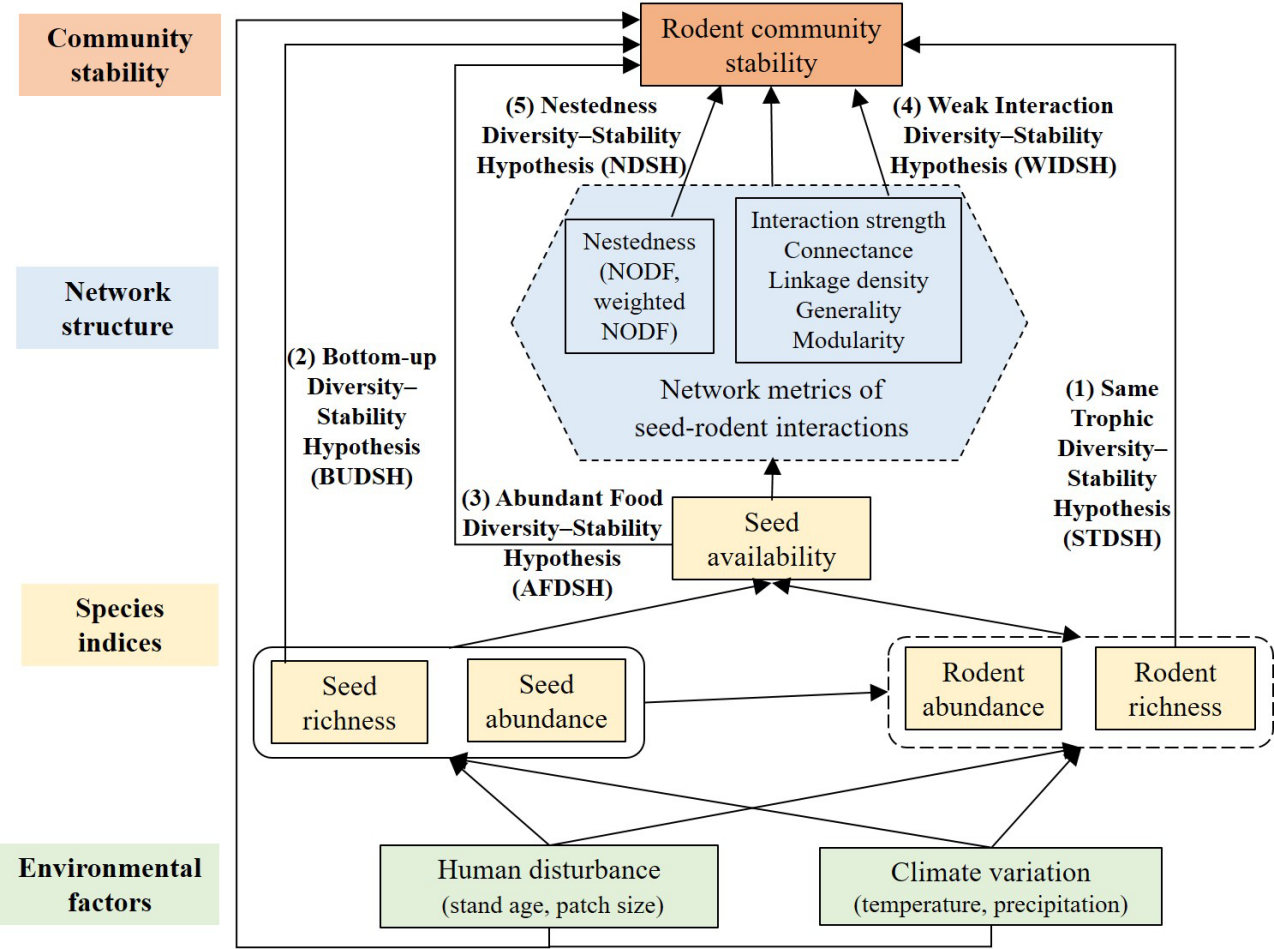
Conceptual framework on the potential direct and indirect relationships between rodent community stability and species richness and abundance of seeds and rodents, the network structure of seed-rodent interactions, and environmental factors. We tested the following hypotheses (1) Rodent community stability should be positively associated with rodent diversity of the same trophic level (Same Trophic Diversity-Stability Hypothesis, STDSH). (2) Rodent community stability should be positively associated with seed diversity due to the bottom-up effect of resource diversity on consumers (Bottom-Up Diversity-Stability Hypotheses, BUDSH). (3) Rodent community stability should be positively associated with seed availability (Abundant Food Diversity-Stability Hypothesis, AFDSH). (4) Rodent community stability should be negatively associated with interaction strength and connectance, or linkage density or generality, or modularity (Weak Interaction Diversity-Stability Hypothesis, WIDSH). (5) Rodent community stability should be positively associated with network nestedness (Nestedness Diversity Stability-Hypothesis, NDSH). Human disturbance and climate variation may have other pathways affecting rodent community stability, e.g., habitats and predators.

By integrating seed tagging and infrared camera trapping methods, we analyzed associations of rodent community stability with species diversity, species abundance, and seed-rodent network complexity under human disturbance and climate variation. We proposed a conceptual framework for testing several key hypotheses (**Figure 1**). We assumed that habitat loss or fragmentation due to human disturbance and climate variation would mainly affect the diversity and abundance of seeds and rodents, and seed availability of rodents; they may affect rodent community stability by affecting the population dynamics of rodents; they may also affect the seed-rodent network structure and then affect rodent community stability. Besides, habitat fragmentation and climate variation may affect rodent community stability by altering habitats or predators of rodents. In this study, we want to test the following hypotheses (**Figure 1**):

(1) Rodent community stability should be positively associated with rodent diversity of the same trophic level (Same Trophic Diversity-Stability Hypothesis, STDSH). Species of the same trophic level are highly complementary to each other as a function group, thus, the community with more species (horizontal diversity) would be less prone to external disturbance (e.g., Mccann et al., 1998; Tilman et al., 2006; Zhao et al., 2019; Downing et al., 2020).

(2) Rodent community stability should be positively associated with seed diversity due to the bottom-up effect of resource diversity on consumers (Bottom-Up Diversity-Stability Hypotheses, BUDSH). Diversified plant species should provide stable food resources to consumers of the same trophic which often share similar food niches.

(3) Rodent community stability should be positively associated with seed availability (Abundant Food Diversity-Stability Hypothesis, AFDSH). Abundant food in seed-rich years or patches should result in seed predator satiation, which may reduce competition among rodent species (Vander Wall, 1990; Xiao et al., 2013); such seed availability-dependent non-monotonic interactions should benefit rodent community stability (e.g., Yan and Zhang, 2014; Zhang et al., 2015; Zeng et al., 2021).

(4) Rodent community stability should be negatively associated with interaction strength and connectance, or linkage density and generality, or modularity (Weak Interaction Diversity-Stability Hypothesis, WIDSH) (e.g., May, 1972; Mccann et al., 1998; Berlow, 1999; Downing et al., 2020).

(5) Rodent community stability should be positively associated with network nestedness (Nestedness Diversity-Stability Hypothesis, NDSH). A nested structure of a network may increase stability by allowing competitors to facilitate each other by sharing mutualistic partners, thereby reducing the negative effects of interspecific competition (e.g., Bascompte and Jordano, 2007; Mougi and Kondoh, 2012).

## Materials and methods

### Study region

We performed this study in a fragmented subtropical broad-leaved evergreen forest (600-1000 m a.s.l., 31°04′ N, 103°43′ E), located in the Dujiangyan region of Sichuan Province, Southwestern China. The Dujiangyan region is a hotspot of biodiversity in China and a priority area for biodiversity conservation. The climate is often cloudy and foggy, with a mean annual temperature of 15.2°C, annual mean sunlight of 800-1000 h, annual precipitation of 1200-l800 mm, and an annual mean relative humidity of 80%. Vegetation is dominated by the Fagaceae species *Quercus serrata* Thunb., *Quercus variabilis* Blume, *Castanopsis fargesii* Franch., *Quercus glauca* Thunb., and the Anacardiaceae species *Choerospondias axillaris* (Roxb.) Burtt & Hill and *Toxicodendron vernicifluum* (Stokes) F. A. Barkley. The shrubby understory is diverse and rich in the Theaceae species *Camellia* sp. (L., 1753) and the Symplocaceae species *Symplocos sp* (Jacq., 1760). Seed rains of those trees vary greatly across seasons and years (Yang et al., 2020), and their seeds are consumed and/or hoarded by several rodent species (Chang and Zhang, 2014; Yang et al., 2018). Common rodent species include *Apodemus draco* (Barrett-Hamieton, 1900), *Niviventer fulvescens* (Gray, 1847), *Niviventer confucianus* (Milne-Edwards, 1891), *Leopoldamys edwardsi* (Thomas, 1882), and *Apodemus chevrieri* (Milne-Edwards, 1868) (Yang et al., 2018; Yang et al., 2022b).

### Sampling plot

In this study, we focused on studies of commonly seen plant seeds that mainly depend on seed dispersal or predation by rodents and seed-rodent interaction network structure, and their responses to human disturbance. We used patch size (fragmentation effect) and stand age (deforestation effect) to represent the intensity of human disturbance. Our study was conducted annually in 15 forest patches with various patch sizes and stand ages from 2013 to 2021. Most of the forests were cleared from 1980 to 2005, and subsequently, forest fragments were allowed to recover on hilltops while lowlands were used for cultivation or road construction by people. Patch size ranged from 2.68 to 57.51 ha (21.68 ± 18.49 ha, mean ± SD) (**Table S1**). The stand age represented the time of recovery after deforestation by local residents for timber and firewood. These forest patches were classified into three categories (i.e., young, middle, and old stands) based on stand age and the degree of human disturbance (detailed see: **Figure S1**; **Table S1**). We used stand age and three category groups for further analysis.

### Quantifying seed and rodent species composition

We collected fallen seeds using seed traps made of Vinylon (New Agricultural Net Factory, Dujiangyan, China, mesh size = 2 mm, sampled area 1 × 1 m) every 2 weeks from early August to late December when seeds matured each year (Yang et al., 2018). Sampling effort for each patch was roughly proportional to patch size, resulting in two transects on a small patch and four transects on a large patch, 3-7 seed traps with a spacing of 10 m between adjacent traps were placed in a patch (detailed see: Yang et al., 2018). In August 2013, we set up 134 traps suspended 0.8 m above the ground using bamboo or trunk posts in 10 patches. We added 44 traps to the other 4 patches in August 2015, and 11 traps to another patch in August 2020.

We used wired live traps (30 × 13 × 12 cm) to capture rodents from October to November before seed dispersal experiments per patch per year. We placed them into 4 × 10 grids with intervals of 10 m in each plot for 5 consecutive nights (200 trap nights). Traps were placed in the afternoon, baited with fresh chestnuts (a food favored by rodents), and were checked the next morning. All captured animals were weighed and identified according to species, sex, and reproductive status. Different species were also marked with distinguishable patterns (such as “|”, “+”, “—”, etc.) on their back with wine-red human hair dye (Gu et al., 2017; Yang et al., 2018) and then released *in situ*. Color and pattern labeling was used to estimate abundance and identify species on infrared camera traps and determine if they harvested (ate or removed) released seeds at the seed stations. Procedures for capturing and handling animals were in accordance with the regulations of the Institute of Zoology, Chinese Academy of Sciences.

As no single index can capture all the characteristics of species assembly, we considered multiple species indices for testing the impacts of human disturbance or climate variation as well as their associations with network metrics and community stability. Species richness of seeds (SR) and rodents (RR) was measured as the number of species observed in each patch. Seed abundance (SA) was measured as the relative density of seeds produced by a patch. Rodent abundance (RA) was estimated using the minimum number alive (MNA) per 100 trap nights by the live traps method described above by a patch (Xiao et al., 2013; Yang et al., 2018). Species diversity was calculated with the Shannon-Weiner index (Hill, 1973):


(1) $$H'=\sum_{i}^{S}P_{i}\text{ln}(P_{i})$$

where *S* = the number of species observed, *P*_*i*_ is the proportion of the sample represented by species *i*. The species diversity index of rodents and seeds is abbreviated as RSDI (rodent Shannon diversity index) and SSDI (seed Shannon diversity index). Furthermore, due to the energetic value of different seed species and the body mass of different rodent species that varied greatly, we also calculated the metabolic seed abundance (estimated by the seed calorific value per seed species, MSA, i.e., seed biomass) and metabolic rodent abundance (the sum of the metabolic-scaling body mass from each rodent species each year, MRA, i.e., rodent biomass).


(2) $$\text{MSA}=\sum_{i=1}^{S}n_{i}CV_{i}$$

(3) $$\text{MRA}=\sum_{i=1}^{S}n_{i}BM_{i}^{0.75}$$

where *S* = the number of seed or rodent species; *n*_*i*_ the number or MNA of a given seed or rodent species *i*; *CV*_*i*_ the average calorific value of a given seed species *i*; $BM_{i}^{0.75}$ the average metabolic-scaling body mass of a given rodent species *i*. Metabolic per capita seed availability (MPCSA): MPCSA = MSA/MRA (Yang et al., 2018), and this index was used to refer to seed availability. The rodent abundance of the following year were used to examine the relationship with climate variation because climate-driven seeds often have a 1-yr delay effect on rodent abundance (Flowerdew et al., 2017). The growth rate of rodent abundance (GR) was defined as GR= (the rodent abundance of the following year—the rodent abundance of the current year)/the rodent abundance of the current year × 100%.

### Quantifying seed-rodent interactions

Seed removal trials were conducted from early November 2013 to mid-January 2022. We used plastic tags with different shapes representing various individual seeds and placed infrared camera traps (Jansen et al., 2012) to record individual seeds harvested by animals, which enabled us to measure the strength of interaction between rodents and tree seeds, as following Yang et al. (2018). We selected seeds of 10 common tree species: *Q. serrata*, *Q. variabilis*, *Q. acutissima* Carruth, *Q. glauca*, *Lithocarpus hancei* (Benth.) Rehder, *L. harlandii* (Hance ex Walp.) Rehder, *C. fargesii*, *C. ceratacantha* Rehder & E. H. Wilson, *Choerospondias axillaris*, and *Camellia oleifera* C. Abel. As the *C. axillaris* seeds were harvested less by rodents, we replaced them with *Castanea mollissima* Blume seeds in 2021. The seed rain periods of these plant seeds generally overlapped with only some variations in peak time (Yang et al., 2020). During the peak period of seed rain, fresh and intact seeds of each species were collected from the ground and/or trees outside of the experimental patch and then air-dried in a cool place.

In each patch, 1-9 seed species were released to monitor rodents harvesting seeds. The number of seeds was decided based on the seed availability of each patch, except for 2013 when seeds from all patches were used. Before seed release, a 0.3-0.4 mm diameter hole was drilled through the husk near the germinal disc of each seed using a portable electric drill. The seeds were tied with small, light white plastic tags (3.6 × 2.5 cm,< 0.1 g) through the hole using a 10 cm long thin steel wire; each tag was coded with a serial number using a marker pen to identify every seed (Yang et al., 2018). Each species included 10 tagged seeds with unique codes that matched different tag shapes, spaced evenly on the soil surface within 1–2 m^2^. A released station contains 10-90 seeds, and each patch repeated 9 released stations. A total of 90–810 tagged seeds were released in a patch per year. After seed release, infrared camera traps (Ltl-5210A or Ltl-5310W; Zhuhai Ltl Acorn Electronics Co., Ltd, Zhuhai, China) were tied to tree trunks adjacent to each seed station (40-70 cm above ground) and set on video record mode (Video size: 640 × 480; PIR sensitivity: High; Video length: 20 s; Trigger interval: 0 s) to monitor seeds harvested by rodents for 3–8 days (according to this research more than 60% of released seeds were harvested by rodents). We repeated the same procedures from 2015 to 2021. A total of 47,400 intact seeds were randomly selected from 11 tree seeds for conducting seed-rodent interaction experiments. We systematically analyzed the video recordings (all capacity 2,702 gigabytes) and identified rodent (combining species morphological traits, color and pattern labeling; Gu et al., 2017; Yang et al., 2018) and seed species for each interaction in the laboratory (**Table S1**).

### Estimating seed-rodent network metrics

Among various network metrics, we selected only 8 network-level metrics that are widely used for studying network structure and stability (Bascompte and Jordano, 2007) in the bipartite package (Dormann et al., 2008): (1) Interaction strength (IS), which is a direct measure of seeds visited by rodents, calculated as IS = the overall number of seeds harvested by rodents divided by the total number of tagged seeds released × 100% (Zhao et al., 2016; Yang et al., 2018). (2) Connectance, the proportion of realized/possible links in a network (Dunne et al., 2002). (3) Nestedness, which describes the tendency for specialist nodes of one type to interact with generalist nodes of the other type, such that more specialist nodes interact with a subset of the nodes that more generalized nodes are connected with; we used a standard measure of nestedness (NODF and weighted NODF) (Almeida-Neto et al., 2008; Montesinos-Navarro et al., 2017), which are more consistent and “better” than usual measures of nestedness. (4) Modularity, quantified whether interactions in each patch formed distinct modules; we used the DIRTLPAwb + algorithm for maximizing modularity (Beckett, 2016) to identify groups of seed-rodent interactions that interacted more strongly within than among modules. (5) Linkage density, which is the number of links divided by the number of species (Tylianakis et al., 2007). (6) Generality, the weighted mean number of host (i.e., seed) species per parasitoid (i.e., rodent) (Tylianakis et al., 2007). (7) Interaction strength asymmetry, which quantifies the balance of effects and influences among species that interact (Bascompte et al., 2006).

### Assessing rodent community stability

We used temporal variability of community to assess community stability by following Thebault and Loreau (2005). Temporal variability is commonly measured as the coefficient of variation (CV, here calculated as standard deviation/mean ×100%) (Thebault and Loreau, 2005; Mcwilliams et al., 2019). Thus, the temporal variability of community is inversely related to community stability; higher temporal variability of the community indicates lower community stability. For our study, temporal variability of the rodent community was measured as the CV of rodent abundance and biomass over 9 consecutive years of 15 patches. A lower CV of rodent abundance and biomass suggested higher rodent community stability.

### Statistical analysis

Among the 110 seed-rodent networks of 15 patches over 8 years, 20 sub-networks were only one-to-many and were excluded from further analysis because many metrics, such as nestedness and modularity were meaningless and could not be calculated. Linear mixed models (LMMs) were used to test the significant effects of stand age or patch size as a fixed factor on species indices, network metrics, and community stability across 15 patches, with year as a random factor. In general, forests with greater human disturbance have smaller patch areas and stand ages (due to shorter succession time), two statistical models were conducted to minimize the effects of collinearity of stand age and patch size in the model analysis. In Model 1, stand age was used as a fixed factor, and year as a random factor; in Model 2, patch size was used as a fixed factor, and year as a random factor; the model selection followed the lowest Akaike information criteria (AICc). LMMs were also used to detect the significant effects of species indices on network metrics and community stability, with year as a random factor. As species indices are inter-dependent (e.g., SR, SA, and MSA are correlated seed indices), we only considered model formulations (here y indicates response variable) like y = f(RR, SR), f(RA, SA), f(MRA, MSA), or f(MPCSA) to avoid collinearity of closely related variables in models by following Yang et al. (2018). Before analysis, we used log- or sqrt-transformed to transform variables to reduce skewness and to normalize the residuals, if necessary. Spearman correlation analysis was used to examine the correlations between the network metrics and rodent community stability. General linear models were used to detect the significant effects of seed species indices on the growth rate of rodent abundance, the rodent abundance of the following year, and network metrics in different stands.

We used Patefield null models (Dormann et al., 2008) to test whether the network metrics across the study plots were significantly different from chance. To achieve this, all corresponding metrics were simulated 1,000 times each and null model-based metrics were compared with observed values (**Table S2**).

To detect the effects of climate variation on seed species indices, rodent abundance, the growth rate of rodent abundance and network metrics, we obtained the climate data of the Dujiangyan region during 2012–2021, including temperature and precipitation from the China Meteorological Data Service Center (CMDC; http://data.cma.cn ), which is publicly accessible. We first calculated the monthly average air temperature (°C), average maximum air temperature (°C), average minimum air temperature (°C), and the monthly accumulative precipitation (mm). Since trees of the 11 seeds species used in the experiment mostly bore fruit from June to July, and the climate had a lag effect on seed dropping phenology, the average temperature (Tem_mean), average maximum temperature (Tem_max), average minimum temperature (Tem_min) and average cumulative precipitation in the first three months (April through June; **Figure S2**) of seed dropping phenology were then calculated and used for subsequent analysis (see similar analysis in Menzel et al. (2006)). General linear models were used to determine the effects of climate factors on seed species indices, the rodent abundance of the following year, the growth rate of rodent abundance, and network metrics.

In addition, because the LMMs assume that species diversity and network metrics and/or abiotic effects are additive, but not interactive, we further constructed models *via* confirmatory path analysis, a type piecewise SEM (Shipley, 2009; Lefcheck, 2016) to infer the relative importance of stand age, species indices and network metrics on the CV of rodent abundance. We constructed the piecewise SEM based on our conceptual path diagram and independent model results, by using variables with VIF (variance inflation factor)< 3 to select variables for the model analyses. Specifically, we hypothesized that the relationship between the CV of rodent abundance and stand age would be mediated by species diversity and network metrics.

The analysis was performed by packages vegan, bipartite, maxnodf, lme4, lmerTest, wiqid, car, ggplot2, ggpubr, Rmisc, and *piecewiseSEM* in R version 4.2.1 (R Core Team, 2022).

## Results

### Association of species diversity and abundance with human disturbance and climate variation

**Figure 2:**
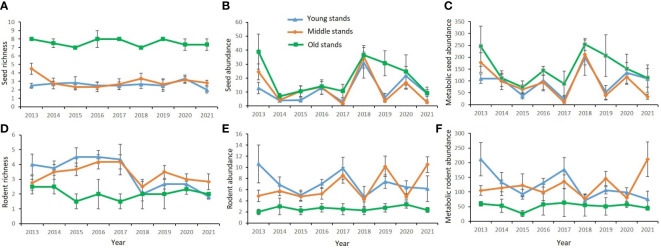
Dynamics of species richness and abundance (biomass) of seeds and rodents in different stands from 2013 to 2021. **(A)** seed richness; **(B)** seed abundance; **(C)** metabolic seed abundance; **(D)** rodent richness; **(E)** rodent abundance; **(F)** metabolic rodent abundance.

**Figure 3:**
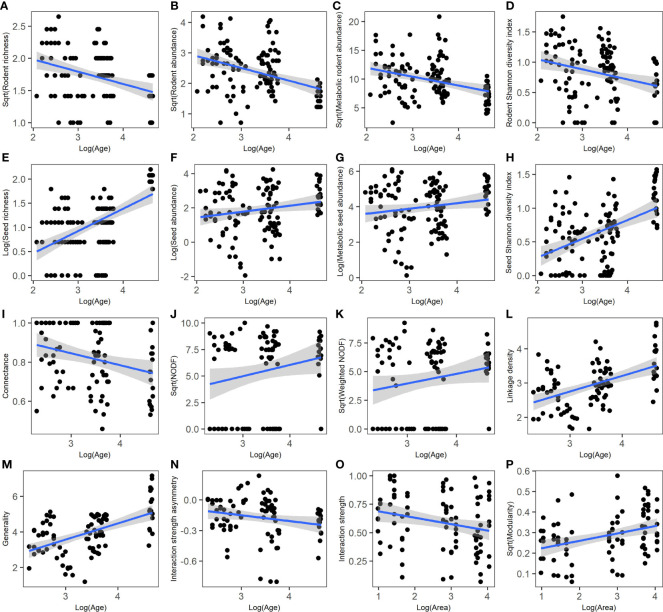
Relationships between stand age (or patch size) and species indices **(A–H)** and network metrics at the community level **(I–P)**. **(A)** rodent richness; **(B)** rodent abundance; **(C)** metabolic rodent abundance, **(D)** rodent Shannon diversity index; **(E)** seed richness; **(F)** seed abundance; **(G)** metabolic seed abundance; **(H)** seed Shannon diversity index; **(I)** connectance; **(J)** nestedness (NODF); **(K)** weighted NODF; **(L)** linkage density; **(M)** generality; **(N)** interaction strength asymmetry; **(O)** interaction strength; **(P)** modularity. Regression lines (with 95% confidence bands) indicate there is a significant relationship between the two variables (*P*< 0.05) based on linear mixed models (**Supplemental Table S3**).

During the 9 consecutive years, seed richness (SR) ranged from 1 to 9 species, seed abundance (SA) ranged from 0.14 to 68.88 No./m^2^, and seed biomass (MSA) ranged from 1.13 to 442.33 KJ across the 15 forest patches; rodent richness (RR) ranged from 1 to 7 species, rodent abundance (RA) ranged from 0.5 to 17.5 individuals per 100 live traps, and rodent biomass (MRA) ranged from 5.79 to 434.84 g across the 15 forest patches. SR, SA, and MSA were significantly higher in old stands than in young and middle stands, while RR, RA, and MRA were significantly higher in young and middle stands than in old stands; the species indices of seeds and rodents showed less fluctuation in old stands than in young and middle stands (**Figure 2**). Statistical analysis showed that the model fit performance as to the effects of stand age on species indices is better than that of patch size through model selection (lowest AICc) (**Table S3**). Stand age had a significant and negative association with rodent species indices (**Figures 3A–D**; **Table S3**), but a significant and positive association with seed species indices (**Figures 3E–H**; **Table S3**). Stand age had a significant and positive association with seed availability (MPCSA; **Table S3**).

From 2012 to 2021, the air temperature and precipitation in the Dujiangyan region showed obvious fluctuation between years, with the highest temperature and rainfall in 2018 (**Figure S2**). Precipitation and temperature had a significant and positive association with seed abundance and seed biomass, but no significant association with seed richness (**Figure S3**; **Table S4**). Further analysis for the three stands showed that SA had a significant and positive association with temperature and precipitation in all stands; MSA had a significant and positive association with precipitation in all stands, but a significant and positive association with temperature only in the middle stands; SR had no significant association with temperature and precipitation in all stands (**Table S5**).

Precipitation and temperature showed a significant and positive association with the growth rate of rodent abundance and the rodent abundance of the following year (**Figure S3**; **Table S6**). Further analysis for the three stands showed that the growth rate of rodent abundance showed a significant and positive association with temperature and precipitation in young and middle stands, but not in old stands; the rodent abundance of the following year showed a significant and positive association with temperature and precipitation only in middle stands (**Table S7**).

MPCSA had a significant and positive association with the growth rate of rodent abundance in young and middle stands, but not in old stands; SR had no significant association with the growth rate of rodent abundance in three stands (**Figure S4**; **Table S8**). In addition, MPCSA had a significant and positive association with the rodent abundance of the following year in young and middle stands, but a significant and negative association with the rodent abundance of the following year in old stands; SR had no significant association with the rodent abundance of the following year in all stands. SA had a significant and positive association with the growth rate of rodent abundance only in middle stands, and a significant and positive association with the rodent abundance of the following year only in young and middle stands (**Table S8**).

### Association of seed-rodent network metrics with human disturbance and climate variation

**Figure 4:**
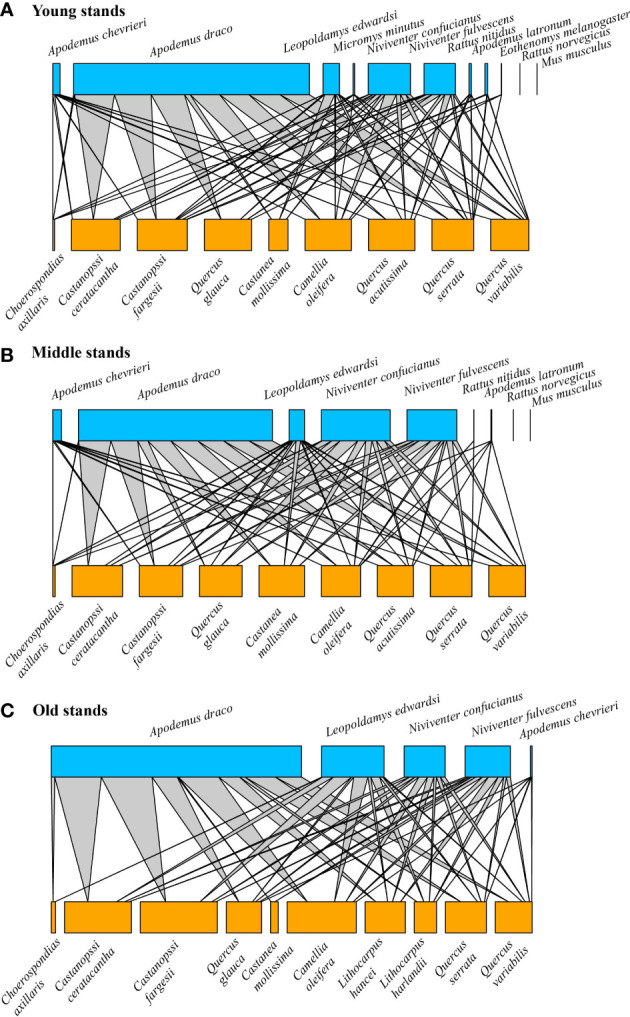
Pooled seed-rodent interaction networks from three stands covering 6, 6, and 3 patches, respectively, over 8 consecutive years during 2014–2021. **(A)** Young stands, **(B)** Middle stands, and **(C)** Old stands. The size of the blue square (top) and orange square (bottom) indicated the relative abundance of the rodent and seed interaction, respectively. The grey line indicated the interaction between rodents and seeds, and the line thickness indicated the interaction strength.

In the 15 forest patches for 8 consecutive years (from 2014 to 2021, except for 2013 because seed placement differed from the subsequent years), we reconstructed 110 seed-rodent networks, which were composed of 26,307 individual harvested events representing 1,131 unique links between 11 tree seed species and 9 rodent species. The seed-rodent networks exhibited an intermediate nestedness (NODF = 43.614) and low modularity (0.093) with high connectance (0.819). Seed-rodent networks had significantly higher nestedness (Z-scores > 2, P< 0.01) and lower modularity (Z-scores< -2, P< 0.01) than that in most null models (**Table S2**). Older stands contained more seed species but fewer rodent species, and lower connectance but higher nestedness (**Figure 4**). When the bipartite graphs were presented separately by year, the results were similar to those of the pooled networks from the three stands (**Figure S5**).

According to the results of best-fitting linear mixed models, stand age showed a significant and negative association with connectance and interaction strength asymmetry, but a significant and positive association with NODF, weighted NODF, linkage density, and generality (**Figures 3I–N**; **Table S3**). Patch size showed a significant and positive association with modularity, but a significant and negative association with interaction strength (**Figures 3O, P**; **Table S3**).

Temperature had a significant and negative association with interaction strength and connectance, and a significant and positive association with nestedness (NODF or weighted NODF), but no significant association with other network metrics; precipitation had no significant association with the network metrics (**Table S9**).

### Association of seed-rodent network metrics with species diversity and abundance

Nestedness (NODF or Weighted NODF) and modularity showed a significant and negative association with rodent abundance (biomass), but a significant and positive association with MPCSA. Linkage density and generality had a significant and positive association with seed richness, seed abundance (biomass) and MPCSA. Interaction strength and connectance showed a significant and positive association with rodent abundance (biomass), and connectance showed a significant and negative association with MPCSA (**Table S10**). Further analysis for the three stands showed that MPCSA had a significant and positive association with linkage density and generality in young and middle stands, but no significant association with linkage density or a significant and positive association with generality in old stands (**Figure S6**; **Table S11**).

### Association of rodent community stability with species diversity, abundance, network metrics, and environmental factors

**Figure 5:**
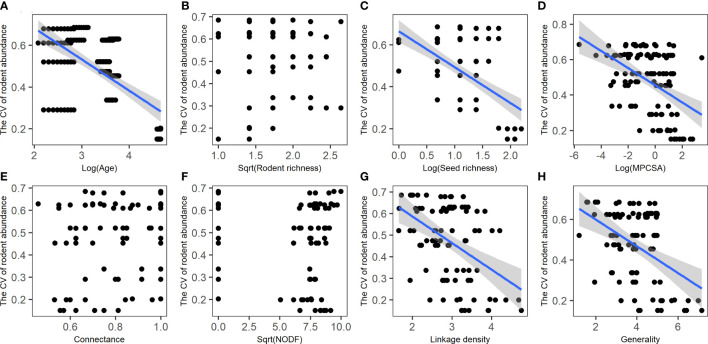
Relationships of rodent community stability (i.e., CV of rodent abundance) with stand age **(A)**, rodent diversity **(B)**, seed diversity **(C)**, MPCSA **(D)**, and seed-rodent network metrics **(E–H)**. MPCSA, metabolic per capita seed availability. Regression lines (with 95% confidence bands) indicate there is a significant relationship between the two variables (*P*< 0.05) based on linear mixed models or general linear models (**Supplemental Tables S3**, **S10**; **Figure S7**).

Human disturbance affects inter-annual changes in rodent abundance (biomass) mainly *via* stand age rather than patch size (**Table S3**). The CV of rodent abundance (biomass) significantly decreases with increasing stand age (**Figure 5A**; **Table S3**), and had a significant and negative association with seed richness, seed abundance (biomass), and seed availability, but no significant association with none of the rodent species indices (**Figures 5B–D**; **Table S10**). The CV of rodent abundance (biomass) had a significant and negative association with linkage density and generality, but no significant association with other network metrics (**Figures 5E–H**; **S7**).

### Synthesis models of bottom-up effects on rodent community stability

**Figure 6:**
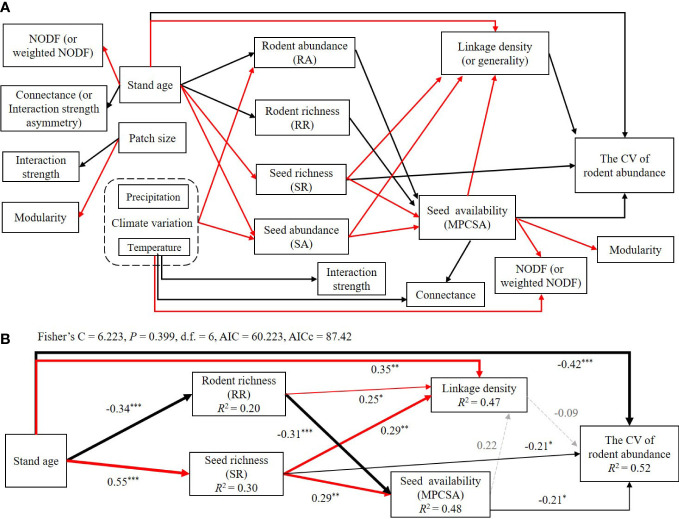
Summary of all significant associations among the CV of rodent abundance, species richness, species abundance, network metrics, and climatic and environmental factors **(A)**, and results of structural equation models (SEM) of stand age on the CV of rodent abundance *via* species diversity and network metrics **(B)**. In panel **(A)**, solid red and black arrows represent the significant and positive or negative associations between the two variables and the potential paths, respectively (**Supplemental Tables S3–S11**; **Figure S7**). In panel **(B)**, solid red arrows represent the significant and positive relationship or potential paths (*P<* 0.05 (*),< 0.01(**),< 0.001(***), piecewise SEM), solid black arrows represent the significant and negative relationship or potential paths (*P<* 0.05 (*),< 0.01(**),< 0.001(***)), and dotted gray arrows represent non-significant paths (*P* > 0.05). Arrow size is proportional to the relative value of the coefficient for each pairwise relationship. We report the path coefficients as standardized effect sizes (**Table S12**). The overall fit of piecewise SEM was evaluated using Shipley’s test of d-separation: Fisher’s C statistic (if *P* > 0.05, then no paths are missing and the model is a good fit) and Akaike information criterion (AICc).

Based on the independent model results in the first four sections of the Results, here we summarized the significant associations of environmental factors, species richness and abundance of seeds and rodents, seed availability of rodents, seed-rodent network metrics, and rodent community stability in **Figure 6A**. In summary, higher seed richness and seed availability, higher linkage density and generality, and older stand age significantly and directly reduced the CV of rodent abundance or increased rodent community stability.

By using the SEM model, the model with Fisher’s C statistic P > 0.05 and AICc = 87.42 was selected and explained 52% of the variation in the CV of rodent abundance (**Figure 6B**; **Table S12**). The SEM results further verified the key pathways affecting rodent community stability via seed richness, rodent richness, and seed availability during a period of human disturbance, but the pathways via linkage density (or generality), rodent abundance, and seed abundance to rodent community stability disappeared (**Figure 6B**). In the SEM model analysis, we removed variables with high correlation coefficients (including generality and linkage density) and climate variables (because there was only one time series for climate for all 15 patches, we were not able to calculate its effects on the CV of rodent abundance).

## Discussion

In this study, by using 9 years of data for 15 patches in a subtropical forest, we identified factors and potential pathways affecting rodent community stability at trophic levels based on data of species richness, species abundance, seed availability, and seed-rodent network metrics. We found rodent community stability had a significant and positive association with seed richness, seed availability, linkage density, generality and stand age; stand age and climate could affect rodent community stability via their effects on species richness, species abundance and seed availability, which then would affect rodent community stability by altering the network metrics of seed-rodent interactions. Our results highlighted the significant role of resource diversity and availability on consumers’ community stability experiencing human disturbance and climate variation, and the need to conserve or restore forest ecosystems to stabilize the ecosystem structure and function.

### Relationship between species diversity and stability

It is widely believed that higher biodiversity facilitates community stability (e.g., Mccann et al., 1998; Tilman et al., 2006; Zhao et al., 2019; Downing et al., 2020). However, a few studies have contradicting observations (e.g., Gross et al., 2014; Ma et al., 2017). Ma et al. (2017) found that the temporal stability of plant community biomass is not influenced by plant species diversity in an alpine grassland community. Gross et al. (2014) reported that species richness has a minimal effect on community stability in algal communities. Danet et al. (2021) found a significant and negative association between the stability of community biomass and species richness in stream fish communities. In this study, we found that rodent community stability is not significantly correlated with rodent species richness, which does not support the Same Trophic Diversity–Stability Hypothesis (STDSH). However, we found rodent community stability is significantly correlated with seed richness, supporting the Bottom-Up Diversity–Stability Hypothesis (BUDSH). We provided evidence that resource diversity is also a stabilizing force for community stability of consumers. This is likely that rodents compete with each other indirectly *via* seed resources which may weaken the complementary effects between them (see below).

The stability of food webs may be jointly influenced by both horizontal (within a trophic level) and vertical diversity (number of trophic levels), which are driven by competitive interactions and predation, respectively (Duffy et al., 2007). Although horizontal diversity of both producers and consumers could increase the stability of food webs, vertical diversity decreases the stability of food webs (Pimm and Lawton, 1977; Zhao et al., 2019). In this study, we found seed richness showed a significant and positive association with rodent community stability, supporting the Bottom-Up Diversity–Stability Hypothesis (BUDSH). Our results indicate that more trophic levels are not necessary to destabilize the food webs. Seed crops vary greatly due to the influence of human disturbance and climate change in the study area (**Figure 2**; also see: Xiao et al., 2013; Yang et al., 2020). Diversified seed production would provide stable food to rodents, thereby benefiting the robustness or stability of the rodent community. An alternative explanation is that the seed-rodent interaction is not purely predation as modeled in previous studies, instead, it is changeable between mutualism and predation depending on seed availability (Zeng et al., 2021; Zhang et al., 2021b). Mutualism at low density and antagonism at high density may increase network stability (Yan and Zhang, 2014; Zhang et al., 2015).

### Relationship between food abundance and stability

Apart from diversified food items, abundant food resources may facilitate species’ coexistence by providing stable food resources. In this study, we found that rodent community stability is significantly and positively associated with seed availability, supporting the Abundant Food Diversity–Stability Hypothesis (AFDSH). In seed-rich conditions, rodents prefer to cache more seeds, which likely helps rodents deal with food shortages in space and time (Vander Wall, 1990; Zhang et al., 2021a); while in seed-poor conditions, rodents often consume seeds as soon as they find them, and cache small seeds; thus, variation in seed production would cause shift between mutualism and predation for seeds and rodents (Zeng et al., 2021). Besides, there is often a high proportion of cached seeds pilfered by different rodent species, which results in the mutualism between rodents as predicted by the reciprocal pilferage hypothesis (Vander Wall and Jenkins, 2003). Therefore, species interaction could transform from strong competition to weak competition or even mutualism between rodent species under conditions with high seed availability; such a non-monotonic interaction has been proven to promote species coexistence (Yan and Zhang, 2014; Zhang et al., 2015; Zhang et al., 2021b).

### Relationship between network structure and stability

Recent studies suggest that network structure may influence community stability (Montesinos-Navarro et al., 2017; Downing et al., 2020; Danet et al., 2021). As predicted by May (1972) and the weak interaction hypothesis (Berlow, 1999), more species, with strong or more connectance between species, would result in a less stable community. Other studies showed that food webs tend to be characterized by many weak interactions and few strong ones, indicating that weak interactions are important for food web persistence (Neutel et al., 2002; Downing et al., 2020). Similarly, Ratzke et al. (2020) recently found that stronger interactions reduced the stability of microbial communities. Linkage density provides an assessment of the generalization of the overall network (Tylianakis et al., 2007). Species with high generality are the key nodes of the ecological network and the driving force of community evolution, which is beneficial to the stability and coevolution of the ecological network (Thompson, 2005). Higher linkage density and generality may increase the robustness and stability of the community as predicted by the redundancy hypothesis (Naeem and Li, 1997). In this study, we did not find a direct and significant correlation between rodent community stability and interaction strength and connectance; instead, we found rodent community stability had a significant and positive association with linkage density or generality, not supporting the Weak Interaction Diversity–Stability Hypothesis (WIDSH), but supporting the species redundancy hypothesis. This is likely because the stabilizing mechanism in our system is mainly determined by the food security (both diversity and abundance) for consumer species. High linkage density and generality represent more diversified links of rodents with seeds of various plant species, which may explain the observation that resource diversity is beneficial to consumers’ stability. Different from the assumption of linear species interaction in previous models, the relationship of species interaction in our system was more likely non-monotonic in seed-rich or seed-poor conditions, which allowed more connectance of more species (Zeng et al., 2021; Zhang et al., 2021b). Notably, older stands had a lower connectance and a higher rodent community stability, suggesting weak interaction is still an important driving force for community stability.

Nestedness in mutualistic networks is the tendency for specialists to interact with proper subsets of species interacting with more generalists (Almeida-Neto et al., 2008). As predicted by the nestedness hypothesis (Bascompte and Jordano, 2007; Almeida-Neto et al., 2008), a highly connected and nested architecture promotes community stability for mutualistic networks, whereas the stability of trophic networks is enhanced in compartmented and weakly connected architecture (Thebault and Fontaine, 2010). In this study, we did not find a direct and significant correlation between rodent community stability and nestedness, not supporting the Nestedness Diversity–Stability Hypothesis (NDSH). This is probably because the seed-rodent network is not a simple mutualistic network, but a complex network containing both mutualism and predation interactions (Chang and Zhang, 2014; Yang et al., 2018; Zeng et al., 2021). It is notable that older stands had a higher nestedness and a high rodent community stability, and seed availability had a significant and positive association with nestedness, suggesting nestedness is still an important driving force for a stable community.

### Impacts of human disturbance (stand age, patch size)

Previous studies have shown that, in plant-herbivore and host-parasitoid networks, network structure was altered by habitat modification, and different network properties such as connectance and generality vary depending on the type of interaction network (Tylianakis et al., 2007). But impacts of forest loss or fragmentation on community stability are rarely investigated. In this study, we found that forest succession rather than fragment size had a larger positive or negative impact on species diversity and network structure of seed-rodent interaction. Our results showed that, in older stands, there were fewer rodent species and more seed species, while in younger stands, there are more rodent species and fewer seed species (**Figure 2**). These results are likely because human deforestation reduced the species richness and abundance of plants (seeds) by removing larger trees (Laurance et al., 2002; Liu et al., 2019); while the complex and diverse habitat environment after intermediate human disturbance increased the species richness and abundance of rodents (Duntan and Fox, 1996), and the species composition and diversity of rodents were higher in early successional stages than those in later successional stages (Yang et al., 2018). Our results also showed that, as compared with younger stands, nestedness and linkage density and generality were larger but connectance and interaction strength asymmetry were smaller in older stands; rodent community stability was higher in older stands. Besides, we found in younger stands, linkage density and generality significantly increased with seed availability, but only generality increased with seed availability in old stands. Our results suggested that forest succession after deforestation appeared to increase community stability because the abundant food resources (i.e., more seeds and higher seed availability) in older stands increased food security and caused predator satiation effects, which helped to stabilize rodent communities (**Figure 6**).

### Impacts of climate change

Climate change is known to affect regional weather patterns and phenology (Rafferty et al., 2020) and then causes mismatches and potential losses in the plant-pollinator interactions (Richman et al., 2020), but its effect on the community stability of consumers by altering plant diversity and abundance is not known. In this study, we found that temperature and precipitation had a significant and positive association with seed abundance (biomass), the rodent abundance of the following year, and the growth rate of rodent abundance; the intensity of influence was higher in young and middle stands than in old stands, which provided a good explanation why rodent populations in old stands are more resistant to climate variation. Furthermore, our results showed that the increase of temperature was beneficial to nestedness, but decreased interaction strength and connectance, and vice versa. These results suggested that a warm and wet climate may enhance rodent community stability via increasing food availability. Our results indicated that climate variation was the external driving force to rodent community stability by altering the seed/rodent richness or abundance, but climate effects were more evident in younger stands than in older stands. The environmental conditions in younger stands tended to be more open and full of light, while older stands were more closed and stable. As the stands developed, the microclimate within changed to a shadier, cool and wet environment, and community fluctuation decreased while stability increased (Bazzaz, 1996; Yu et al., 2011).

### Implications for forest management

Previous studies suggest that more pests or diseases often outbreak in monoculture forests or human-disturbed forests (Patz et al., 2004; Dirzo et al., 2014). We found rodent populations fluctuated more noticeably in younger stands than in older stands, which often imposed heavy damage on seed regeneration or reforestation projects, and provide strong evidence that a disturbing system with poor diversity is less stable and prone to pest or disease outbreaks. The forests in this study region experienced different degrees of logging and cultivation for agricultural use from 1980 to 2005, and many large Fagaceae tree species such as *Q. variabilis* and *C. fargesii* were cut, forming many fragmented patches of different sizes and different stages of succession. Therefore, to facilitate the restoration process in degraded forests and maintain the stability of forest ecosystems, it is necessary to protect old-growth forests by reducing human disturbance (such as logging and farming) and facilitating forest succession. In years or young stands with low seed abundance, or with a high abundance of rodents, it is necessary to supplement seeds or control rodents to stabilize the ecosystem structure and function.

Our results provide a novel insight into the relationship between diversity and stability under climate variation and human disturbance. Long-term monitoring and assessment of seed-rodent interaction are needed to reveal the ecological mechanism of network structure in the maintenance of diversity–stability in forest ecosystems under accelerated human disturbances and global climate change.

## Data availability statement

The datasets presented in this study can be found in online repositories. The names of the repository/repositories and accession number(s) can be found below: Data (Yang et al., 2022a) are available on Science DB: https://doi.org/10.57760/sciencedb.j00001.00471 .

## Ethics statement

The animal study was conducted in line with the guidelines of Animal Care and Use Committee of Institute of Zoology, Chinese Academy of Sciences (CAS).

## Author contributions

ZZ and XY designed the research. XY, HG, QZ, YZ, YT, and YL collected the field data. XY performed the mathematical analysis and wrote the first draft of the manuscript. All authors contributed to the article and approved the submitted version.

## Funding

This study was supported by the Strategic Priority Research Program of the Chinese Academy of Sciences (XDB11050300); the National Key Research and Development Program (2017YFC0503802, 2016YFC0500105); the National Natural Science Foundation of China (31330013, 32001123); the China Postdoctoral Science Foundation (2019M650840).

## Acknowledgments

We are very grateful to Xunlong Wang, Chengqiang Wang and Kunming Zhao for field assistance; Institute of Botany, CAS, and Forest Bureau of the Dujiangyan City for field support; Prof. Zhishu Xiao for valuable comments; Chuan Yan, Guoliang Li, Xinru Wan, Haidong Li, and Mingqiang Wang for technical support.

## Conflict of interest

The authors declare that the research was conducted in the absence of any commercial or financial relationships that could be construed as a potential conflict of interest.

## Publisher’s note

All claims expressed in this article are solely those of the authors and do not necessarily represent those of their affiliated organizations, or those of the publisher, the editors and the reviewers. Any product that may be evaluated in this article, or claim that may be made by its manufacturer, is not guaranteed or endorsed by the publisher.
