# Supplementary material for: High seed diversity and availability increase rodent community stability under human disturbance and climate variation
Source: Front Plant Sci. 2022 Nov 30;13:1068795. doi: 10.3389/fpls.2022.1068795 (PMC9748286; doi:10.3389/fpls.2022.1068795)
Supplement: Supplementary file 1 [file DataSheet_1.docx]

**Supplementary material**

**Title:** High seed diversity and availability increase rodent community stability under human disturbance and climate variation

**Authors:** Xifu Yang^1^, Haifeng Gu^1^, Qingjian Zhao^1^, Yunlong Zhu^1,2^, Yuwei Teng^1,2^, Ying Li^1,2^, Zhibin Zhang^1,2*^

^1^State Key Laboratory of Integrated Management of Pest Insects and Rodents in Agriculture, Institute of Zoology, Chinese Academy of Sciences, Beijing 100101, China

^2^CAS Center for Excellence in Biotic Interactions, University of Chinese Academy of Sciences, Beijing 100049, China

^*^**Corresponding author**, Email: zhangzb@ioz.ac.cn

**Table S1** Description of the 15 studied patches and the sampling of seed-rodent interactions over 8 consecutive years in the Dujiangyan region of Sichuan Province, Southwestern China. These forest patches were classified into three categories based on stand age and the degree of human disturbance: (1) Young stands (< 20 years), which had experienced extensive logging and destruction in those 20 years; (2) Middle stands (20–40 years), which also experienced extensive logging and destruction in the early period, and after more than 20 years of vegetation succession, there was a secondary succession stage due to the implementation of government management measures such as prohibiting deforestation, fire prevention initiatives, and other measures; (3) Old stands (> 90 years) experienced less logging and disturbance because these patches were protected by the nearby Banruosi temples. We used specific stand age (only 2013 was shown) for analysis as required in this table. According to the availability of seed species, 1–9 seed species were released to monitor rodents harvesting seeds in each patch. Each species included 10 tagged seeds with unique codes that match different tag shapes, spaced evenly on the soil surface within 1–2 m^2^. A released station contains 10–90 seeds, and each patch repeated 9 released stations. A total of 90–810 tagged seeds were released in a patch per year.

| Patch code | Stand ages  (year)^§^ | Stand age  (2013) | Patch size  (ha.) ^§^ | Numbers of released seed species | | | | | | | |
| --- | --- | --- | --- | --- | --- | --- | --- | --- | --- | --- | --- |
|  |  |  |  | 2014 | 2015 | 2016 | 2017 | 2018 | 2019 | 2020 | 2021 |
| A | Young stand | 9 | 2.68 | 1 | 3 | 3 | 4 | 4 | 4 | 4 | 4 |
| B1 | Old stand | 98 | 40.12 | 6 | 7 | 8 | 9 | 8 | 9 | 8 | 8 |
| B2 | Old stand | 98 | 45.34 | 6 | 8 | 8 | 9 | 8 | 9 | 8 | 7 |
| B3 | Old stand | 98 | 38.62 | – | – | – | – | – | – | 6 | 6 |
| C | Young stand | 9 | 4.20 | 2 | 4 | 4 | 4 | 5 | 6 | 5 | 5 |
| D | Young stand | 9 | 22.99 | 1 | 6 | 4 | 5 | 6 | 6 | 5 | 4 |
| F | Middle stand | 36 | 17.63 | 1 | 6 | 5 | 6 | 6 | 6 | 5 | 5 |
| H | Middle stand | 31 | 6.05 | 1 | 5 | 5 | 5 | 5 | 6 | 5 | 5 |
| K | Middle stand | 28 | 57.51 | 2 | 6 | 6 | 5 | 6 | 6 | 5 | 5 |
| L | Young stand | 8 | 41.89 | 1 | 5 | 5 | 4 | 6 | 6 | 5 | 4 |
| M | Middle stand | 36 | 5.23 | 2 | 4 | 5 | 5 | 5 | 6 | 5 | 5 |
| R | Young stand | 15 | 20.23 | – | 2 | 4 | 3 | 3 | 3 | 2 | 2 |
| S | Middle stand | 28 | 16.18 | – | 4 | 4 | 4 | 4 | 4 | 3 | 4 |
| U | Young stand | 13 | 2.85 | – | 5 | 4 | 4 | 4 | 4 | 3 | 2 |
| V | Middle stand | 33 | 3.75 | – | 4 | 4 | 3 | 4 | 4 | 4 | 4 |
| Total number of released seeds  Total sample sizes (Infrared camera traps) | | | | 1950 | 6210 | 6210 | 6300 | 6660 | 7110 | 6570 | 6300 |
|  |  |  |  | 59GB | 167 GB | 311 GB | 367 GB | 410 GB | 368 GB | 575GB | 445GB |

§ The data of stand age (year) and patch size (ha.) come from Yang et al. (2018) and this study.

**Table S2** Results from *r2dtable* null model testing the significance of the metrics calculated for the seed-rodent interaction networks. For each null model, we generated a set of 1000 randomized networks and compared the distribution of network metrics to the observed value (Dormann et al., 2008). *r2dtable* null model generates a set of random 2-way tables with given marginal totals using Patefield’s algorithm (Yodzis, 1981). This null model constrains the marginal totals to be identical to those observed, i.e., the number of harvests is fixed for each seed-rodent interaction. Thus, the information is preserved that some seed-rodent interactions occur more frequently than others. Results showed that, when compared with the *r2dtable* null model, the quantitative seed-rodent interaction networks showed a significant nested and weak modular pattern.

| Year | Patch | Connectance | | | Nestedness (NODF) | | | Modularity | | |
| --- | --- | --- | --- | --- | --- | --- | --- | --- | --- | --- |
|  |  | Observed value | Mean of  null model | Z-score | Observed value | Mean of  null model | Z-score | Observed value | Mean of  null model | Z-score |
| 2014 | B2 | 0.600 | 0.722 | -11.428 | 46.154 | 72.446 | 39.689 | 0.237 | 0.127 | -3.002 |
| 2014 | C | 1.000 | 1.000 | NA | 0.000 | 0.000 | NA | 0.117 | 0.029 | -1.212 |
| 2014 | K | 0.667 | 0.942 | -10.260 | 75.000 | 23.625 | 74.322 | 0.212 | 0.052 | -1.785 |
| 2015 | A | 0.667 | 0.949 | -11.271 | 75.000 | 22.350 | 74.349 | 0.068 | 0.020 | -1.966 |
| 2015 | B1 | 0.750 | 0.883 | -26.002 | 62.346 | 51.404 | 58.248 | 0.211 | 0.075 | -4.267 |
| 2015 | B2 | 0.583 | 0.650 | -18.679 | 37.500 | 52.275 | 30.682 | 0.034 | 0.026 | -5.430 |
| 2015 | C | 0.813 | 0.977 | -28.164 | 62.500 | 18.008 | 61.762 | 0.208 | 0.061 | -3.081 |
| 2015 | D | 1.000 | 0.951 | -18.017 | 0.000 | 22.500 | -0.990 | 0.067 | 0.037 | -2.537 |
| 2015 | F | 0.458 | 0.776 | -15.487 | 69.048 | 73.467 | 60.558 | 0.100 | 0.032 | -3.874 |
| 2015 | H | 0.550 | 0.823 | -15.033 | 75.000 | 67.915 | 68.466 | 0.091 | 0.034 | -3.527 |
| 2015 | K | 0.600 | 0.876 | -11.655 | 45.455 | 45.473 | 43.228 | 0.116 | 0.038 | -2.667 |
| 2015 | L | 0.550 | 0.761 | -23.415 | 81.250 | 78.146 | 68.512 | 0.267 | 0.085 | -3.154 |
| 2015 | M | 0.625 | 0.763 | -8.890 | 57.143 | 65.429 | 47.868 | 0.071 | 0.060 | -3.247 |
| 2015 | S | 0.625 | 0.625 | NA | 57.143 | 57.143 | NA | 0.041 | 0.045 | -9.629 |
| 2016 | B1 | 0.750 | 0.872 | -25.377 | 69.785 | 45.891 | 66.139 | 0.170 | 0.072 | -3.808 |
| 2016 | B2 | 0.531 | 0.696 | -18.923 | 52.941 | 72.073 | 45.520 | 0.181 | 0.059 | -3.654 |
| 2016 | C | 0.917 | 0.993 | -41.332 | 55.556 | 4.500 | 55.259 | 0.075 | 0.038 | -2.744 |
| 2016 | D | 0.833 | 0.833 | NA | 55.556 | 55.556 | NA | 0.090 | 0.054 | -2.575 |
| 2016 | F | 0.733 | 0.862 | -51.608 | 84.615 | 47.815 | 77.743 | 0.097 | 0.043 | -2.776 |
| 2016 | H | 0.700 | 0.836 | -34.305 | 93.750 | 51.469 | 89.988 | 0.235 | 0.062 | -3.278 |
| 2016 | K | 0.733 | 0.851 | -21.409 | 82.000 | 65.792 | 76.337 | 0.163 | 0.063 | -4.021 |
| 2016 | L | 0.950 | 0.967 | -30.792 | 43.750 | 24.956 | 42.633 | 0.108 | 0.059 | -3.834 |
| 2016 | M | 0.533 | 0.654 | -11.816 | 76.923 | 77.441 | 69.315 | 0.047 | 0.026 | -3.068 |
| 2016 | R | 1.000 | 0.947 | -14.240 | 0.000 | 25.771 | -0.904 | 0.070 | 0.019 | -2.033 |
| 2016 | S | 1.000 | 0.989 | -32.573 | 0.000 | 6.511 | -0.364 | 0.083 | 0.032 | -2.808 |
| 2016 | U | 0.875 | 0.942 | -14.117 | 57.143 | 25.257 | 56.256 | 0.066 | 0.018 | -2.125 |
| 2016 | V | 1.000 | 1.000 | NA | 0.000 | 0.000 | NA | 0.113 | 0.050 | -3.092 |
| 2017 | A | 1.000 | 1.000 | NA | 0.000 | 0.000 | NA | 0.094 | 0.048 | -2.532 |
| 2017 | B1 | 0.708 | 0.824 | -20.323 | 74.194 | 61.869 | 67.500 | 0.057 | 0.037 | -3.953 |
| 2017 | B2 | 0.963 | 0.958 | -33.945 | 25.641 | 24.562 | 23.803 | 0.191 | 0.062 | -4.164 |
| 2017 | C | 1.000 | 1.000 | NA | 0.000 | 0.000 | NA | 0.059 | 0.047 | -3.058 |
| 2017 | D | 1.000 | 1.000 | NA | 0.000 | 0.000 | NA | 0.061 | 0.033 | -2.258 |
| 2017 | F | 0.800 | 0.986 | -33.843 | 61.538 | 7.846 | 61.088 | 0.119 | 0.046 | -3.246 |
| 2017 | H | 0.900 | 0.936 | -18.590 | 45.455 | 29.682 | 44.083 | 0.020 | 0.035 | -2.475 |
| 2017 | K | 0.800 | 0.914 | -21.386 | 76.923 | 42.785 | 73.509 | 0.046 | 0.046 | -3.163 |
| 2017 | L | 1.000 | 0.996 | -38.852 | 0.000 | 1.425 | -0.139 | 0.038 | 0.022 | -1.859 |
| 2017 | M | 0.833 | 0.951 | -17.348 | 66.667 | 28.978 | 65.659 | 0.062 | 0.034 | -2.943 |
| 2017 | R | 0.667 | 0.751 | -12.275 | 88.889 | 74.172 | 83.709 | 0.122 | 0.067 | -2.090 |
| 2017 | S | 0.850 | 0.890 | -20.877 | 75.000 | 52.942 | 68.135 | 0.067 | 0.065 | -3.609 |
| 2017 | U | 0.750 | 0.749 | -11.877 | 55.556 | 75.594 | 50.201 | 0.138 | 0.047 | -2.256 |
| 2017 | V | 0.917 | 0.993 | -41.786 | 55.556 | 4.056 | 55.275 | 0.081 | 0.054 | -2.853 |
| 2018 | A | 0.625 | 0.710 | -11.558 | 57.143 | 65.814 | 47.916 | 0.011 | 0.008 | -2.733 |
| 2018 | B1 | 0.571 | 0.619 | -14.664 | 83.951 | 77.281 | 75.445 | 0.073 | 0.030 | -3.915 |
| 2018 | B2 | 0.844 | 0.930 | -30.537 | 70.588 | 41.095 | 67.470 | 0.166 | 0.061 | -4.444 |
| 2018 | C | 0.800 | 0.877 | -24.806 | 61.538 | 48.631 | 55.558 | 0.062 | 0.032 | -3.104 |
| 2018 | D | 1.000 | 1.000 | NA | 0.000 | 0.000 | NA | 0.061 | 0.047 | -2.596 |
| 2018 | F | 0.733 | 0.906 | -17.116 | 84.615 | 48.874 | 81.756 | 0.053 | 0.028 | -3.142 |
| 2018 | K | 0.958 | 0.923 | -34.368 | 38.095 | 40.810 | 20.793 | 0.087 | 0.053 | -4.351 |
| 2018 | L | 0.792 | 0.831 | -27.483 | 76.190 | 63.836 | 71.400 | 0.188 | 0.064 | -3.975 |
| 2018 | R | 1.000 | 0.966 | -13.147 | 0.000 | 14.025 | -0.479 | 0.142 | 0.038 | -1.788 |
| 2018 | S | 0.833 | 0.820 | -25.854 | 55.556 | 59.989 | 50.592 | 0.066 | 0.061 | -2.403 |
| 2018 | V | 0.750 | 0.770 | -12.636 | 55.556 | 70.972 | 50.325 | 0.071 | 0.049 | -2.138 |
| 2019 | B1 | 0.556 | 0.798 | -15.670 | 69.231 | 64.030 | 60.747 | 0.075 | 0.036 | -4.352 |
| 2019 | C | 0.667 | 0.705 | -13.577 | 56.250 | 58.713 | 44.575 | 0.008 | 0.007 | -4.546 |
| 2019 | D | 0.750 | 0.702 | -13.681 | 62.500 | 58.372 | 51.519 | 0.008 | 0.009 | -3.919 |
| 2019 | F | 0.700 | 0.793 | -11.128 | 63.636 | 60.455 | 54.890 | 0.013 | 0.008 | -2.987 |
| 2019 | H | 0.600 | 0.600 | NA | 45.455 | 45.455 | NA | 0.004 | 0.004 | -239.077 |
| 2019 | K | 0.833 | 0.909 | -30.855 | 66.667 | 41.904 | 58.688 | 0.082 | 0.047 | -3.880 |
| 2019 | L | 0.833 | 0.920 | -24.509 | 65.278 | 38.239 | 61.751 | 0.089 | 0.035 | -3.173 |
| 2019 | M | 0.583 | 0.583 | -110.200 | 37.500 | 37.313 | 23.400 | 0.007 | 0.006 | -9.501 |
| 2019 | R | 0.667 | 0.886 | -12.008 | 100.000 | 57.000 | 97.620 | 0.332 | 0.131 | -2.276 |
| 2019 | S | 1.000 | 0.998 | -70.564 | 0.000 | 0.800 | -0.110 | 0.081 | 0.036 | -2.278 |
| 2019 | U | 0.667 | 0.741 | -12.826 | 88.889 | 79.639 | 82.814 | 0.094 | 0.042 | -2.271 |
| 2019 | V | 1.000 | 1.000 | NA | 0.000 | 0.000 | NA | 0.008 | 0.043 | -3.097 |
| 2020 | A | 1.000 | 0.999 | -75.556 | 0.000 | 0.686 | -0.110 | 0.030 | 0.026 | -2.273 |
| 2020 | B1 | 0.813 | 0.808 | -74.602 | 44.118 | 42.176 | 35.649 | 0.146 | 0.047 | -4.708 |
| 2020 | B2 | 0.844 | 0.951 | -32.576 | 57.647 | 34.389 | 55.485 | 0.136 | 0.050 | -4.465 |
| 2020 | B3 | 0.875 | 0.978 | -37.841 | 57.143 | 19.452 | 56.187 | 0.052 | 0.039 | -3.989 |
| 2020 | C | 1.000 | 1.000 | NA | 0.000 | 0.000 | NA | 0.067 | 0.042 | -3.374 |
| 2020 | D | 0.700 | 0.804 | -16.220 | 81.250 | 72.074 | 74.661 | 0.162 | 0.069 | -3.196 |
| 2020 | F | 1.000 | 1.000 | NA | 0.000 | 0.000 | NA | 0.028 | 0.035 | -2.633 |
| 2020 | H | 1.000 | 0.896 | -11.497 | 0.000 | 41.218 | -1.885 | 0.008 | 0.011 | -3.053 |
| 2020 | K | 0.850 | 0.932 | -24.829 | 56.250 | 43.719 | 53.652 | 0.064 | 0.045 | -3.387 |
| 2020 | L | 0.867 | 0.856 | -11.746 | 61.538 | 57.935 | 57.948 | 0.022 | 0.024 | -3.408 |
| 2020 | M | 0.700 | 0.735 | -9.433 | 63.636 | 60.873 | 54.316 | 0.047 | 0.027 | -2.920 |
| 2020 | R | 1.000 | 1.000 | NA | 0.000 | 0.000 | NA | 0.009 | 0.033 | -1.390 |
| 2020 | S | 0.833 | 0.967 | -21.786 | 55.556 | 20.833 | 54.781 | 0.089 | 0.045 | -2.666 |
| 2020 | U | 1.000 | 1.000 | NA | 0.000 | 0.000 | NA | 0.030 | 0.030 | -1.901 |
| 2021 | V | 1.000 | 1.000 | NA | 0.000 | 0.000 | NA | 0.027 | 0.047 | -3.075 |
| 2021 | B1 | 0.625 | 0.838 | -16.602 | 76.882 | 58.870 | 70.550 | 0.154 | 0.073 | -3.765 |
| 2021 | B2 | 0.667 | 0.733 | -21.211 | 47.917 | 52.150 | 43.860 | 0.250 | 0.059 | -2.927 |
| 2021 | B3 | 1.000 | 0.994 | -44.164 | 0.000 | 2.288 | -0.255 | 0.109 | 0.044 | -2.775 |
| 2021 | F | 1.000 | 1.000 | NA | 0.000 | 0.000 | NA | 0.028 | 0.033 | -2.700 |
| 2021 | K | 0.950 | 0.946 | -25.552 | 43.750 | 37.413 | 41.919 | 0.066 | 0.054 | -3.746 |
| 2021 | L | 1.000 | 0.969 | -15.576 | 0.000 | 14.757 | -0.587 | 0.048 | 0.038 | -2.311 |
| 2021 | M | 1.000 | 0.959 | -16.095 | 0.000 | 19.291 | -0.816 | 0.038 | 0.018 | -2.652 |
| 2021 | R | 1.000 | 1.000 | NA | 0.000 | 0.000 | NA | 0.220 | 0.044 | -1.649 |
| 2021 | S | 1.000 | 0.999 | -140.065 | 0.000 | 0.650 | -0.115 | 0.073 | 0.050 | -3.266 |
| 2021 | U | 1.000 | 1.000 | NA | 0.000 | 0.000 | NA | 0.075 | 0.022 | -1.281 |
| 2021 | V | 1.000 | 1.000 | NA | 0.000 | 0.000 | NA | 0.113 | 0.052 | -3.828 |

**Table S3** Analyses of variance through Wald χ^2^ tests for testing the significance of stand age and patch size from best-fitting linear mixed models with species indices or network metrics as response variables. In general, forests with greater human disturbance have smaller patch areas and shorter succession time, two statistical models were conducted to prevent collinearity of the model, respectively. Model 1 used stand age as a fixed factor, and year as a random factor; model 2 used patch size as a fixed factor, and year as a random factor; model selection followed the lowest AICc. MRA, metabolic rodent abundance; MSA, metabolic seed abundance; MPCSA, metabolic per capita seed availability (MPCSA= MSA/MRA). Temporal variability of community was used to assess community stability, and temporal variability of community is commonly measured as the coefficient of variation (CV, here calculated as standard deviation/mean ×100%) (Thebault and Loreau 2005). Fixed factors in bold indicate the best-fitting model and significant differences (*P* < 0.05). The analysis was performed by the *Anova* function in the *car* package in R software.

| Items | Model 1* | | Model 2* | | Chi-square test | | |
| --- | --- | --- | --- | --- | --- | --- | --- |
|  | *d.f.* | AICc | *d.f.* | AICc | *χ^2^* | *d.f.* | *P* |
| Species indices |  |  |  |  |  |  |  |
| Rodent richness | 4 | **109.089** | 4 | 122.165 | 16.290 | 1 | < 0.001 |
| Rodent abundance | 4 | **262.695** | 4 | 278.603 | 27.090 | 1 | < 0.001 |
| MRA | 4 | **625.272** | 4 | 636.046 | 15.372 | 1 | < 0.001 |
| Rodent Shannon diversity index | 4 | **132.494** | 4 | 140.666 | 9.079 | 1 | 0.003 |
| Seed richness | 4 | **191.675** | 4 | 218.896 | 54.592 | 1 | < 0.001 |
| Seed abundance | 4 | **380.168** | 4 | 384.764 | 7.773 | 1 | 0.005 |
| MSA | 4 | **391.596** | 4 | 395.184 | 6.050 | 1 | 0.014 |
| Seed Shannon diversity index | 4 | **133.224** | 4 | 151.359 | 31.817 | 1 | < 0.001 |
| MPCSA | 4 | **430.835** | 4 | 440.3 | 16.822 | 1 | < 0.001 |
| Network metrics |  |  |  |  |  |  |  |
| Connectance | 4 | **-81.452** | 4 | -68.557 | 16.294 | 1 | < 0.001 |
| NODF | 4 | **489.652** | 4 | 491.181 | 7.851 | 1 | 0.005 |
| Weighted NODF | 4 | **464.362** | 4 | 467.085 | 6.262 | 1 | 0.012 |
| Linkage density | 4 | **-27.085** | 4 | -21.423 | 5.063 | 1 | 0.024 |
| Generality | 4 | **171.654** | 4 | 178.895 | 27.776 | 1 | < 0.001 |
| Interaction strength asymmetry | 4 | **268.428** | 4 | 286.879 | 38.922 | 1 | < 0.001 |
| Interaction strength | 4 | 6.893 | 4 | **5.985** | 6.009 | 1 | 0.014 |
| Modularity | 4 | -124.956 | 4 | **-132.686** | 11.673 | 1 | 0.001 |
| Community stability |  |  |  |  |  |  |  |
| The CV of rodent abundance | 4 | **-116.327** | 4 | -58.226 | 20.034 | 1 | < 0.001 |
| The CV of rodent biomass (MRA) | 4 | **-88.690** | 4 | -76.839 | 7.833 | 1 | < 0.001 |

Model 1 *, response variable ~ stand age + (1|year)

Model 2 *, response variable ~ patch size + (1|year)

**Table S4** Results of general linear model analyses between climatic factors and species richness and abundance (biomass) of seeds in the Dujiangyan region. Fixed factors in bold indicate significant differences (*P* < 0.05).

| Climatic factors | Seed richness (SR) | | | | | Seed abundance (SA) | | | | | Seed biomass (MSA) | | | | |
| --- | --- | --- | --- | --- | --- | --- | --- | --- | --- | --- | --- | --- | --- | --- | --- |
|  | *Estimate* ± *SE* | *d.f.* | *t* | *P* | *R*^2^ | *Estimate* ± *SE* | *d.f.* | *t* | *P* | *R*^2^ | *Estimate* ± *SE* | *d.f.* | *t* | *P* | *R*^2^ |
| Average temperature | 0.004 ± 0.081 | 118 | 0.044 | 0.965 | <0.001 | **0.536** ± **0.171** | **118** | **3.143** | **0.002** | **0.077** | **0.378** ± **0.175** | **118** | **2.160** | **0.033** | **0.038** |
| Average maximum temperature | 0.038 ± 0.090 | 118 | 0.421 | 0.675 | 0.001 | **0.940** ± **0.179** | **118** | **5.249** | **<0.001** | **0.189** | **0.570** ± **0.193** | **118** | **2.960** | **0.004** | **0.069** |
| Average minimum temperature | -0.011 ± 0.065 | 118 | -0.170 | 0.866 | <0.001 | **0.289** ± **0.142** | **118** | **2.039** | **0.044** | **0.034** | 0.268 ± 0.143 | 118 | 1.878 | 0.063 | 0.029 |
| Average cumulative precipitation | 0.098 ± 0.267 | 118 | 0.367 | 0.714 | 0.001 | **3.073** ± **0.515** | **118** | **5.962** | **<0.001** | **0.232** | **3.035** ± **0.520** | **118** | **5.840** | **<0.001** | **0.224** |

**Table S5** Results of general linear model analyses between climatic factors and species richness and abundance (biomass) of seeds in different stands. Fixed factors in bold indicate significant differences (*P* < 0.05).

| Stand ages | Seed species indices | Average maximum temperature | | | | |  | Average cumulative precipitation | | | | |
| --- | --- | --- | --- | --- | --- | --- | --- | --- | --- | --- | --- | --- |
|  |  | *Estimate* ± *SE* | *d.f.* | *t* | *P* | *R*^2^ |  | *Estimate* ± *SE* | *d.f.* | *t* | *P* | *R*^2^ |
| Young stands | Seed richness (SR) | 0.115 ± 0.105 | 48 | 1.100 | 0.277 | 0.025 |  | -0.006 ± 0.310 | 48 | -0.019 | 0.985 | < 0.001 |
|  | Seed abundance (SA) | **0.784** ± **0.321** | **48** | **2.443** | **0.018** | **0.111** |  | **3.012** ± **0.895** | **48** | **3.364** | **0.002** | **0.191** |
|  | Seed biomass (MSA) | 0.416 ± 0.356 | 48 | 1.167 | 0.249 | 0.028 |  | **3.122** ± **0.955** | **48** | **3.267** | **0.002** | **0.182** |
| Middle stands | Seed richness (SR) | 0.044 ± 0.119 | 48 | 0.368 | 0.715 | 0.003 |  | 0.272 ± 0.347 | 48 | 0.786 | 0.436 | 0.013 |
|  | Seed abundance (SA) | **1.300** ± **0.230** | **48** | **5.639** | **< 0.001** | **0.399** |  | **3.612** ± **0.695** | **48** | **5.198** | **< 0.001** | **0.360** |
|  | Seed biomass (MSA) | **0.898** ± **0.245** | **48** | **3.659** | **0.001** | **0.218** |  | **3.420** ± **0.644** | **48** | **5.314** | **< 0.001** | **0.370** |
| Old stands | Seed richness (SR) | -0.014 ± 0.038 | 18 | -0.367 | 0.718 | 0.007 |  | -0.026 ± 0.120 | 18 | -0.213 | 0.834 | 0.003 |
|  | Seed abundance (SA) | **0.583** ± **0.235** | **18** | **2.484** | **0.023** | **0.255** |  | **1.802** ± **0.737** | **18** | **2.444** | **0.025** | 0.249 |
|  | Seed biomass (MSA) | 0.289 ± 0.228 | 18 | 1.266 | 0.222 | 0.082 |  | **1.786** ± **0.614** | **18** | **2.908** | **0.009** | **0.320** |

**Table S6** Results of general linear model analyses between climatic factors and the rodent abundance of the following year and the growth rate of rodent abundance in the Dujiangyan region. Fixed factors in bold indicate significant differences (*P* < 0.05).

| Climatic factors | Rodent abundance of the following year | | | | |  | Growth rate of rodent abundance | | | | |
| --- | --- | --- | --- | --- | --- | --- | --- | --- | --- | --- | --- |
|  | *Estimate* ± *SE* | *d.f.* | *t* | *P* | *R*^2^ |  | *Estimate* ± *SE* | *d.f.* | *t* | *P* | *R*^2^ |
| Average temperature | 0.174 ± 0.138 | 113 | 1.259 | 0.210 | 0.014 |  | **0.929** ± **0.298** | **103** | **3.122** | **0.002** | **0.086** |
| Average maximum temperature | **0.276** ± **0.127** | **113** | **2.169** | **0.032** | **0.040** |  | **1.490** ± **0.294** | **103** | **5.059** | **< 0.001** | **0.199** |
| Average minimum temperature | 0.061 ± 0.112 | 113 | 0.544 | 0.587 | 0.003 |  | 0.454 ± 0.245 | **103** | 1.852 | 0.067 | 0.032 |
| Average cumulative precipitation | **0.886** ± **0.321** | **113** | **2.757** | **0.007** | **0.063** |  | **2.854** ± **0.697** | **103** | **4.096** | **< 0.001** | **0.140** |

**Table S7** Results of general linear model analyses between climatic factors and the rodent abundance of the following year and the growth rate of rodent abundance in different stands. Bold indicate significant differences (*P* < 0.05).

| Stand ages | Rodent species indices | Average maximum temperature | | | | |  | Average cumulative precipitation | | | | |
| --- | --- | --- | --- | --- | --- | --- | --- | --- | --- | --- | --- | --- |
|  |  | *Estimate* ± *SE* | *d.f.* | *t* | *P* | *R*^2^ |  | *Estimate* ± *SE* | *d.f.* | *t* | *P* | *R*^2^ |
| Young stands | Rodent abundance of the following year | 0.047 ± 0.217 | 46 | 0.215 | 0.831 | 0.001 |  | 0.950 ± 0.533 | 46 | 1.782 | 0.081 | 0.065 |
|  | Growth rate of rodent abundance | **2.188** ± **0.608** | **42** | **3.601** | **0.001** | **0.236** |  | **4.294** ± **1.434** | **42** | **2.994** | **0.005** | **0.176** |
| Middle stands | Rodent abundance of the following year | **0.526** ± **0.149** | **46** | **3.524** | **0.001** | **0.213** |  | **1.009** ± **0.400** | **46** | **2.523** | **0.015** | **0.122** |
|  | Growth rate of rodent abundance | **1.243** ± **0.312** | **42** | **3.981** | **< 0.001** | **0.274** |  | **2.221** ± **0.759** | **42** | **2.925** | **0.006** | **0.169** |
| Old stands | Rodent abundance of the following year | 0.095 ± 0.126 | 17 | 0.755 | 0.461 | 0.032 |  | 0.282 ± 0.331 | 17 | 0.851 | 0.407 | 0.041 |
|  | Growth rate of rodent abundance | 0.235 ± 0.134 | 15 | 1.750 | 0.101 | 0.170 |  | 0.572 ± 0.311 | 15 | 1.842 | 0.085 | 0.185 |

**Table S8** Results of general linear model analyses between seed richness, seed abundance, and seed availability with the rodent abundance of the following year and the growth rate of rodent community in different stands. MPCSA, metabolic per capita seed availability. Bold indicate significant differences (*P* < 0.05).

| Stand ages | Rodent species indices | Seed abundance (SA) | | | | |  | Seed availability (MPCSA) | | | | |  | Seed richness (SR) | | | | |
| --- | --- | --- | --- | --- | --- | --- | --- | --- | --- | --- | --- | --- | --- | --- | --- | --- | --- | --- |
|  |  | *Estimate* ± *SE* | *d.f.* | *t* | *P* | *R*^2^ |  | *Estimate* ± *SE* | *d.f.* | *t* | *P* | *R*^2^ |  | *Estimate* ± *SE* | *d.f.* | *t* | *P* | *R*^2^ |
| Young stands | Rodent abundance of the following year | **1.008** ± **0.381** | **42** | **2.643** | **0.011** | **0.143** |  | **0.683** ± **0.325** | **42** | **2.100** | **0.042** | **0.095** |  | -0.071 ± 0.508 | 42 | -0.140 | 0.889 | < 0.001 |
|  | Growth rate of rodent abundance | 0.346 ± 0.246 | 42 | 1.407 | 0.167 | 0.045 |  | **0.635** ± **0.184** | **42** | **3.449** | **0.001** | **0.221** |  | -0.201 ± 0.309 | 42 | -0651 | 0.519 | 0.010 |
| Middle stands | Rodent abundance of the following year | **1.301** ± **0.397** | **42** | **3.277** | **0.002** | **0.204** |  | **1.098** ± **0.365** | **42** | **3.011** | **0.004** | **0.178** |  | -0.196 ± 0.415 | 42 | -0.473 | 0.639 | 0.005 |
|  | Growth rate of rodent abundance | **0.404** ± **0.137** | **42** | **2.956** | **0.005** | **0.172** |  | **0.420** ± **0.119** | **42** | **3.522** | **0.001** | **0.228** |  | -0.026 ± 0.140 | 42 | -0.183 | 0.856 | < 0.001 |
| Old stands | Rodent abundance of the following year | -0.069 ± 0.344 | 15 | -0.202 | 0.843 | 0.003 |  | **-0.520** ± **0.205** | **15** | **-2.535** | **0.023** | **0.300** |  | 0.199 ± 0.354 | 15 | 0.562 | 0.582 | 0.021 |
|  | Growth rate of rodent abundance | 0.127 ± 0.097 | 15 | 1.299 | 0.214 | 0.101 |  | 0.075 ± 0.071 | 15 | 1.062 | 0.305 | 0.070 |  | -0.013 ± 0.107 | 15 | -0.126 | 0.901 | 0.001 |

**Table S9** Results of general linear model analyses between climatic factors and the network metrics of seed-rodent interactions. Bold indicate significant differences (*P* < 0.05).

| Network metrics | Average temperature | | | | | Average maximum temperature | | | | | Average minimum temperature | | | | | Average cumulative precipitation | | | | |
| --- | --- | --- | --- | --- | --- | --- | --- | --- | --- | --- | --- | --- | --- | --- | --- | --- | --- | --- | --- | --- |
|  | *Estimate* ± *SE* | *d.f.* | *t* | *P* | *R^2^* | *Estimate* ± *SE* | *d.f.* | *t* | *P* | *R^2^* | *Estimate* ± *SE* | *d.f.* | *t* | *P* | *R^2^* | *Estimate* ± *SE* | *d.f.* | *t* | *P* | *R^2^* |
| Interaction strength | **-0.114** ± **0.036** | **88** | **-3.167** | **0.002** | **0.102** | -0.062 ± 0.043 | 88 | -1.448 | 0.151 | 0.023 | **-0.104** ± **0.029** | **88** | **-3.587** | **0.001** | **0.128** | -0.063 ± 0.119 | 88 | -0.526 | 0.600 | 0.003 |
| Modularity | 0.026 ± 0.017 | 88 | 1.499 | 0.137 | 0.025 | 0.002 ± 0.020 | 88 | 0.081 | 0.936 | < 0.001 | 0.026 ± 0.014 | 88 | 1.869 | 0.065 | 0.038 | 0.041 ± 0.054 | 88 | 0.761 | 0.449 | 0.007 |
| Connectance | **-0.074** ± **0.024** | **88** | **-3.126** | **0.002** | **0.100** | -0.049 ± 0.028 | 88 | -1.750 | 0.084 | 0.034 | **-0.073** ± **0.019** | **88** | **-3.884** | **< 0.001** | **0.146** | -0.113 ± 0.077 | 88 | -1.466 | 0.146 | 0.024 |
| NODF | **1.941** ± **0.547** | **88** | **3.550** | **0.001** | **0.125** | **1.729** ± **0.638** | **88** | **2.710** | **0.008** | **0.077** | **1.674** ± **0.441** | **88** | **3.793** | **< 0.001** | **0.141** | 2.205 ± 1.814 | 88 | 1.215 | 0.228 | 0.017 |
| Weighted NODF | **1.554** ± **0.470** | **88** | **3.306** | **0.001** | **0.111** | **1.577** ± **0.540** | **88** | **2.918** | **0.004** | **0.088** | **1.230** ± **0.384** | **88** | **3.203** | **0.002** | **0.104** | 1.494 ± 1.551 | 88 | 0.964 | 0.338 | 0.010 |
| Interaction strength  asymmetry | 0.020 ± 0.031 | 88 | 0.639 | 0.524 | 0.005 | 0.022 ± 0.035 | 88 | 0.647 | 0.520 | 0.005 | 0.008 ± 0.025 | 88 | 0.326 | 0.745 | 0.001 | -0.001 ± 0.096 | 88 | -0.006 | 0.995 | < 0.001 |
| Linkage density | -0.092 ± 0.103 | 88 | -0.896 | 0.373 | 0.009 | 0.037 ± 0.117 | 88 | 0.315 | 0.754 | 0.001 | -0.124 ± 0.083 | 88 | -1.494 | 0.139 | 0.025 | -0.077 ± 0.323 | 88 | -0.237 | 0.813 | < 0.001 |
| Generality | -0.179 ± 0.186 | 88 | -0.964 | 0.338 | 0.010 | 0.059 ± 0.212 | 88 | 0.278 | 0.781 | < 0.001 | -0.214 ± 0.150 | 88 | -1.428 | 0.157 | 0.023 | -0.047 ± 0.584 | 88 | -0.081 | 0.936 | < 0.001 |

**Table S10** Statistically significant effects of variables on network metrics and rodent community stability from best-fitting linear mixed models for the seed-rodent interaction networks across 8 consecutive years. The models used year as a random factor; as species indices are inter-dependent (e.g., RR, RA, and MRA are correlated rodent indices), we only considered model formulations (here y indicates response variable) like: y = f(RR, RA), f(SR, SA), f(MRA, MSA), or f(MPCSA) to avoid collinearity in models by following Yang et al. (2018). RR, rodent richness; RA, rodent abundance; MRA, metabolic rodent abundance; SR, seed richness; SA, seed abundance; MSA, metabolic seed abundance; MPCSA, metabolic per capita seed availability (MPCSA= MSA/MRA). The variables were log- or sqrt- transformed to meet assumptions of the statistical models if necessary. Temporal variability of community was used to assess community stability, and temporal variability of community is commonly measured as the coefficient of variation (CV, here calculated as standard deviation/mean ×100%) (Thebault and Loreau 2005). The analysis was performed by the *lme4* package in R software.

| Response variable | Exploratory variable | *Estimate* | *SE* | *d.f.* | *t* | *P* |
| --- | --- | --- | --- | --- | --- | --- |
| Interaction strength | RR | 0.158 | 0.062 | 84.852 | 2.547 | 0.013 |
|  | RA | 0.128 | 0.030 | 83.509 | 4.257 | < 0.001 |
|  | MRA | 0.026 | 0.007 | 82.749 | 3.768 | < 0.001 |
| Connectance | RA | 0.057 | 0.020 | 83.264 | 2.840 | 0.006 |
|  | MRA | 0.012 | 0.005 | 82.642 | 2.680 | 0.009 |
|  | MPCSA | -0.022 | 0.010 | 87.690 | -2.172 | 0.033 |
| NODF | RA | -1.235 | 0.507 | 84.885 | -2.436 | 0.017 |
|  | MRA | -0.244 | 0.115 | 83.911 | -2.113 | 0.038 |
|  | MPCSA | 0.511 | 0.242 | 81.145 | 2.112 | 0.038 |
| Weighted NODF | RA | -1.010 | 0.436 | 85.002 | -2.315 | 0.023 |
|  | MRA | -0.221 | 0.099 | 83.899 | -2.226 | 0.029 |
|  | MPCSA | 0.438 | 0.207 | 78.378 | 2.113 | 0.038 |
| Linkage density | SR | 0.541 | 0.101 | 76.371 | 5.351 | < 0.001 |
|  | SA | 0.166 | 0.055 | 37.305 | 3.020 | 0.005 |
|  | MSA | 0.180 | 0.054 | 58.733 | 3.311 | 0.002 |
|  | MPCSA | 0.125 | 0.042 | 44.100 | 3.011 | 0.004 |
| Generality | SR | 0.997 | 0.172 | 75.528 | 5.787 | < 0.001 |
|  | SA | 0.316 | 0.101 | 54.183 | 3.122 | 0.003 |
|  | MSA | 0.287 | 0.099 | 72.579 | 2.915 | 0.005 |
|  | MPCSA | 0.273 | 0.076 | 70.596 | 3.596 | 0.001 |
| Modularity | RA | -0.066 | 0.014 | 82.102 | -4.825 | < 0.001 |
|  | MRA | -0.008 | 0.003 | 82.055 | -2.309 | 0.023 |
|  | MPCSA | 0.015 | 0.007 | 83.120 | 2.085 | 0.040 |
| The CV of rodent abundance | SR | -0.158 | 0.026 | 87.000 | -6.185 | < 0.001 |
|  | SA | -0.053 | 0.014 | 45.265 | -3.671 | 0.001 |
|  | MSA | -0.064 | 0.013 | 58.886 | -4.744 | < 0.001 |
|  | MPCSA | -0.051 | 0.010 | 39.806 | -4.990 | < 0.001 |
| The CV of rodent biomass (MRA) | SR | -0.076 | 0.029 | 87.000 | -2.652 | 0.010 |
|  | SA | -0.034 | 0.013 | 87.000 | -2.581 | 0.012 |
|  | MSA | -0.040 | 0.013 | 87.000 | -3.138 | 0.002 |
|  | MPCSA | -0.024 | 0.010 | 88.000 | -2.446 | 0.016 |

**Table S11** Results of general linear model analyses between seed availability (MPCSA) and linkage density and generality in different stands. MPCSA, metabolic per capita seed availability. Bold indicate significant differences (*P* < 0.05).

| Stand ages | Fixed factor | Linkage density | | | | |  | Generality | | | | |
| --- | --- | --- | --- | --- | --- | --- | --- | --- | --- | --- | --- | --- |
|  |  | *Estimate* | *SE* | *d.f.* | *t* | *P* |  | *Estimate* | *SE* | *d.f.* | *t* | *P* |
| Young stands | (Intercept) | 0.948 | 0.256 | 33.000 | 3.709 | **<** 0.001 |  | 0.730 | 0.511 | 33.000 | 1.430 | 0.162 |
|  | MPCSA | **1.517** | **0.228** | **33.000** | **6.663** | **< 0.001** |  | **2.397** | **0.455** | **33.000** | **5.266** | **< 0.001** |
| Middle stands | (Intercept) | 1.194 | 0.434 | 36.858 | 2.750 | 0.009 |  | 0.629 | 0.711 | 6.654 | 0.885 | 0.382 |
|  | MPCSA | **1.338** | **0.318** | **34.218** | **4.205** | **< 0.001** |  | **2.490** | **0.529** | **34.847** | **4.711** | **< 0.001** |
| Old stands | (Intercept) | 2.277 | 1.025 | 10.863 | 2.220 | 0.049 |  | 1.704 | 1.498 | 10.100 | 1.137 | 0.282 |
|  | MPCSA | 0.803 | 0.621 | 10.169 | 1.293 | 0.225 |  | **2.204** | **0.900** | **9.193** | **2.449** | **0.036** |

**Table S12** Standardized direct effects from the structural equation models (SEMs) in Fig. 6B of the main text.

| Response | Path | Predictor | *Estimate* | *Std.Error* | *d.f.* | Crit.Value | *P.Value* | Std.Estimate | Significant |
| --- | --- | --- | --- | --- | --- | --- | --- | --- | --- |
| Rodent richness (RR) | ← | Stand age | -0.192 | 0.054 | 84.164 | 12.451 | 0.001 | -0.342 | *** |
| Seed richness (SR) | ← | Stand age | 0.490 | 0.079 | 86.450 | 37.900 | < 0.001 | 0.553 | *** |
| Seed availability (MPCSA) | ← | Rodent richness (RR) | -1.359 | 0.363 | 84.113 | 13.786 | < 0.001 | -0.313 | *** |
| Seed availability (MPCSA) | ← | Seed richness (SR) | 0.800 | 0.219 | 81.662 | 13.246 | 0.001 | 0.291 | ** |
| Linkage density | ← | Stand age | 0.323 | 0.098 | 80.757 | 10.775 | 0.002 | 0.347 | ** |
| Linkage density | ← | Rodent richness (RR) | 0.414 | 0.167 | 83.458 | 5.966 | 0.017 | 0.250 | * |
| Linkage density | ← | Seed richness (SR) | 0.307 | 0.113 | 80.306 | 7.290 | 0.009 | 0.292 | ** |
| Linkage density | ← | Seed availability (MPCSA) | 0.084 | 0.044 | 82.546 | 3.330 | 0.072 | 0.219 |  |
| The CV of rodent abundance | ← | Stand age | -0.104 | 0.023 | 84.878 | 19.218 | < 0.001 | -0.419 | *** |
| The CV of rodent abundance | ← | Seed richness (SR) | -0.059 | 0.028 | 84.343 | 4.353 | 0.040 | -0.209 | * |
| The CV of rodent abundance | ← | Seed availability (MPCSA) | -0.022 | 0.009 | 32.516 | 5.588 | 0.024 | -0.214 | * |
| The CV of rodent abundance | ← | Linkage density | -0.025 | 0.024 | 84.920 | 1.019 | 0.316 | -0.092 |  |


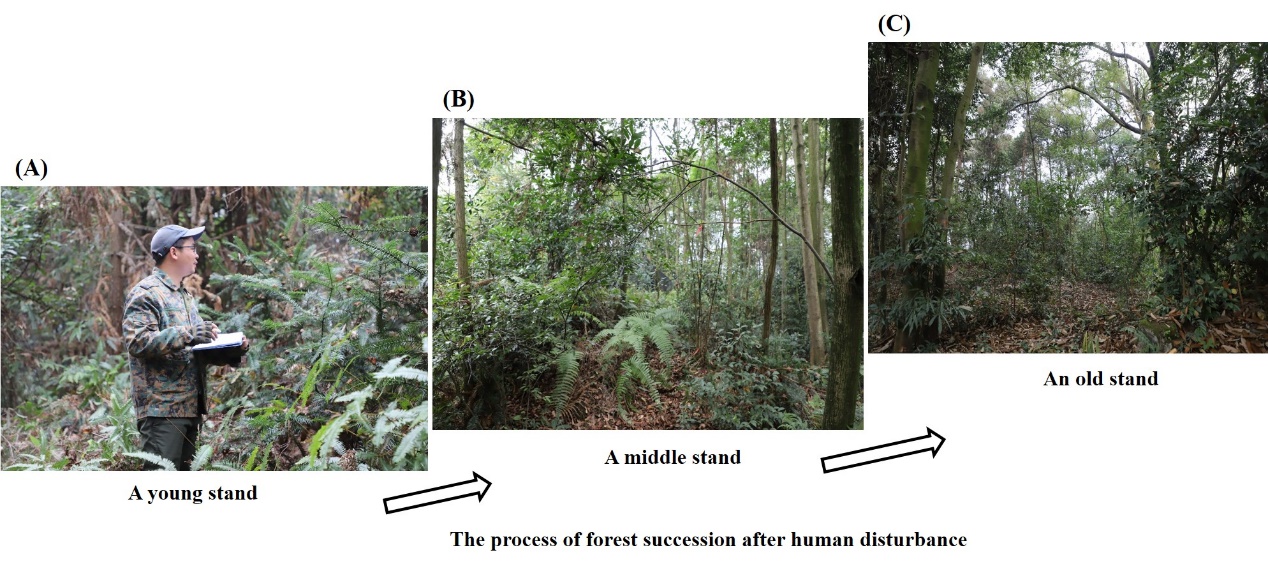


**Figure S1** A schematic diagram of different forest successional stages after human disturbance. (A) Young stands (< 20 years), which had experienced extensive logging and destruction in those 20 years. Young stands often have an abundance of herbs, but few hard mast (acorns, nuts) trees. (B) Middle stands (20–40 years), which also experienced extensive logging and destruction in the early period, and after more than 20 years of vegetation succession, there was a secondary succession stage due to the implementation of government management measures such as deforestation prohibition, fire prevention, and other measures. Middle stands often have an abundance of shrubs and small trees. (C) Old stands (> 90 years) experienced less logging and disturbance because these patches were protected by the nearby Banruosi temples. Old stands often have abundant and tall vegetation and few herbs.


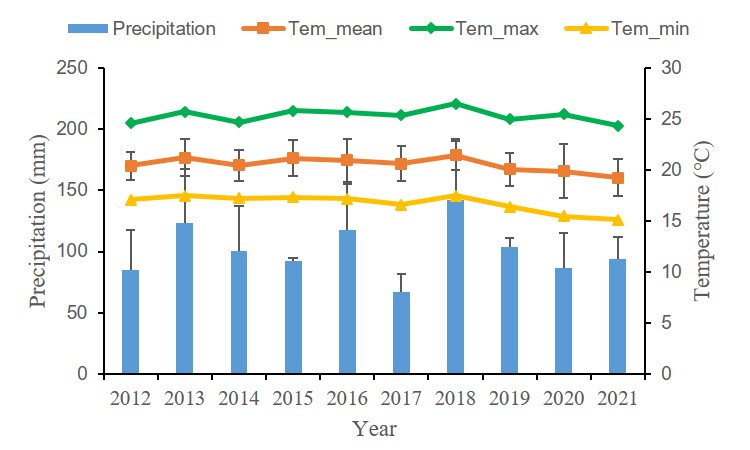


**Figure S2** Changes of average temperature and precipitation for three months (4-6 months) in the Dujiangyan region from 2012 to 2021. We first calculated the monthly average air temperature (℃), average maximum air temperature (℃), average minimum air temperature (℃), and the monthly accumulative precipitation (mm). Since trees of the 11 seeds species used in the experiment mostly bore fruit from June to July, and the climate had a lag effect on seed dropping phenology, the average temperature (Tem_mean), average maximum temperature (Tem_max), average minimum temperature (Tem_min) and average cumulative precipitation in the first three months (April through June**)** of seed dropping phenology were then calculated and used for subsequent analysis (see similar analysis in Menzel et al., 2006). Data are expressed as the mean ± SE.


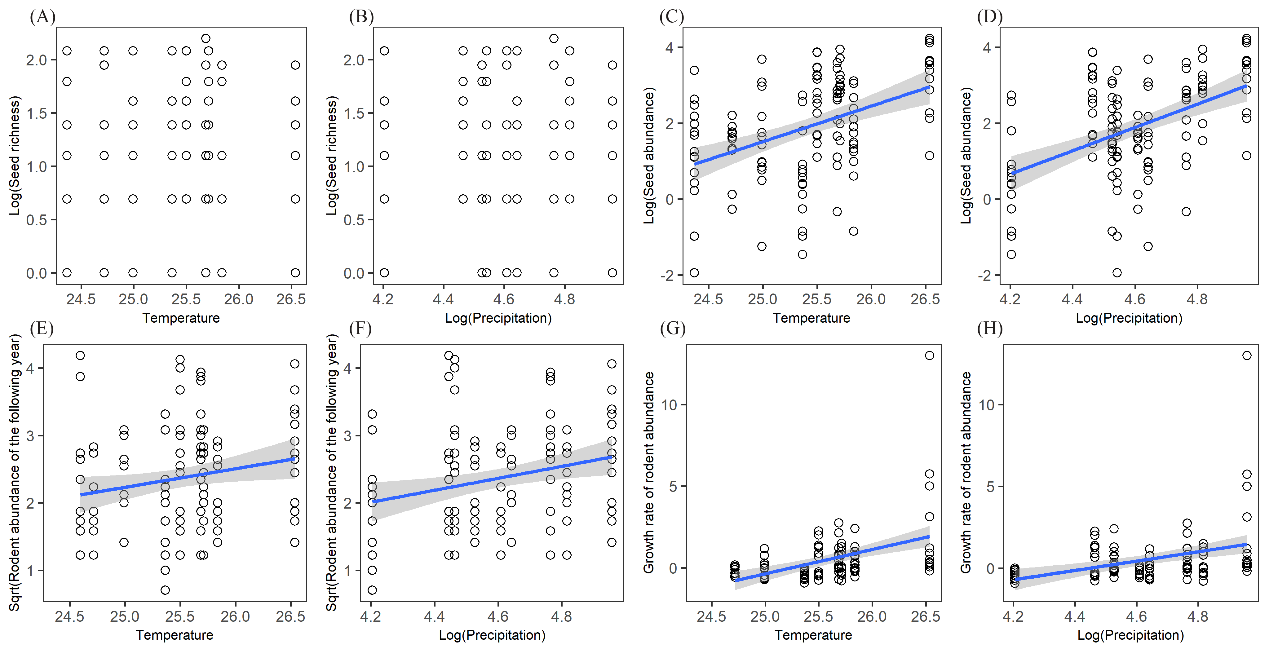


Figure S3 Relationships between climatic factors (average maximum temperature and average cumulative precipitation) and seed richness (A, B), seed abundance (C, D), the rodent abundance of the following year (E, F), and the growth rate of rodent abundance (G, H). Regression lines (with 95% confidence bands) indicate there is a significant relationship between the two variables (*P* < 0.05) based on general linear models (Tables S4 and S6).


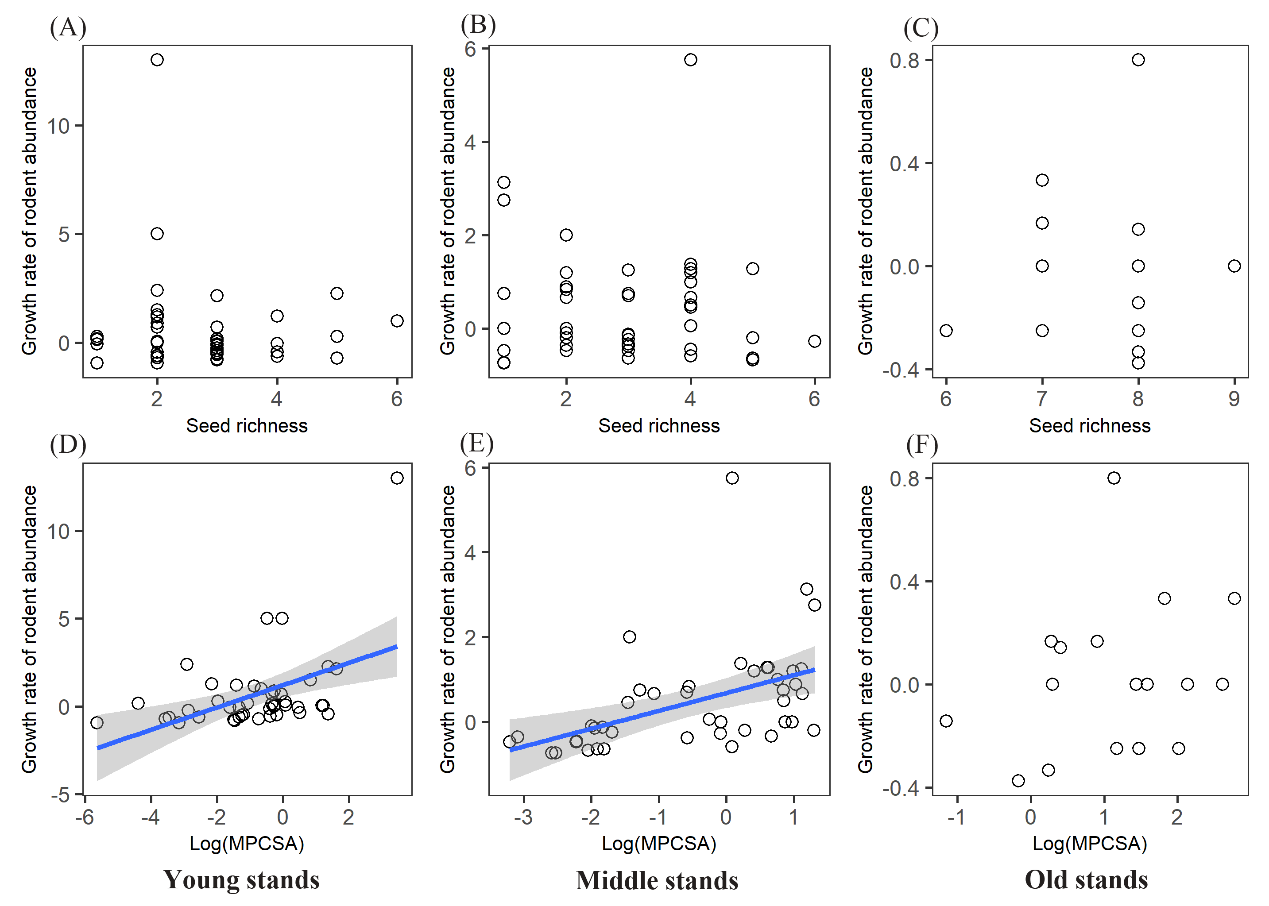


Figure S4 Relationships between the growth rate of rodent abundance and seed richness (A–C) and MPCSA (D–F). The left showed young stands, the middle showed middle stands, and the right showed old stands. MPCSA, metabolic per capita seed availability. Regression lines (with 95% confidence bands) indicate there is a significant relationship between the two variables (*P* < 0.05) based on general linear models (Table S8).


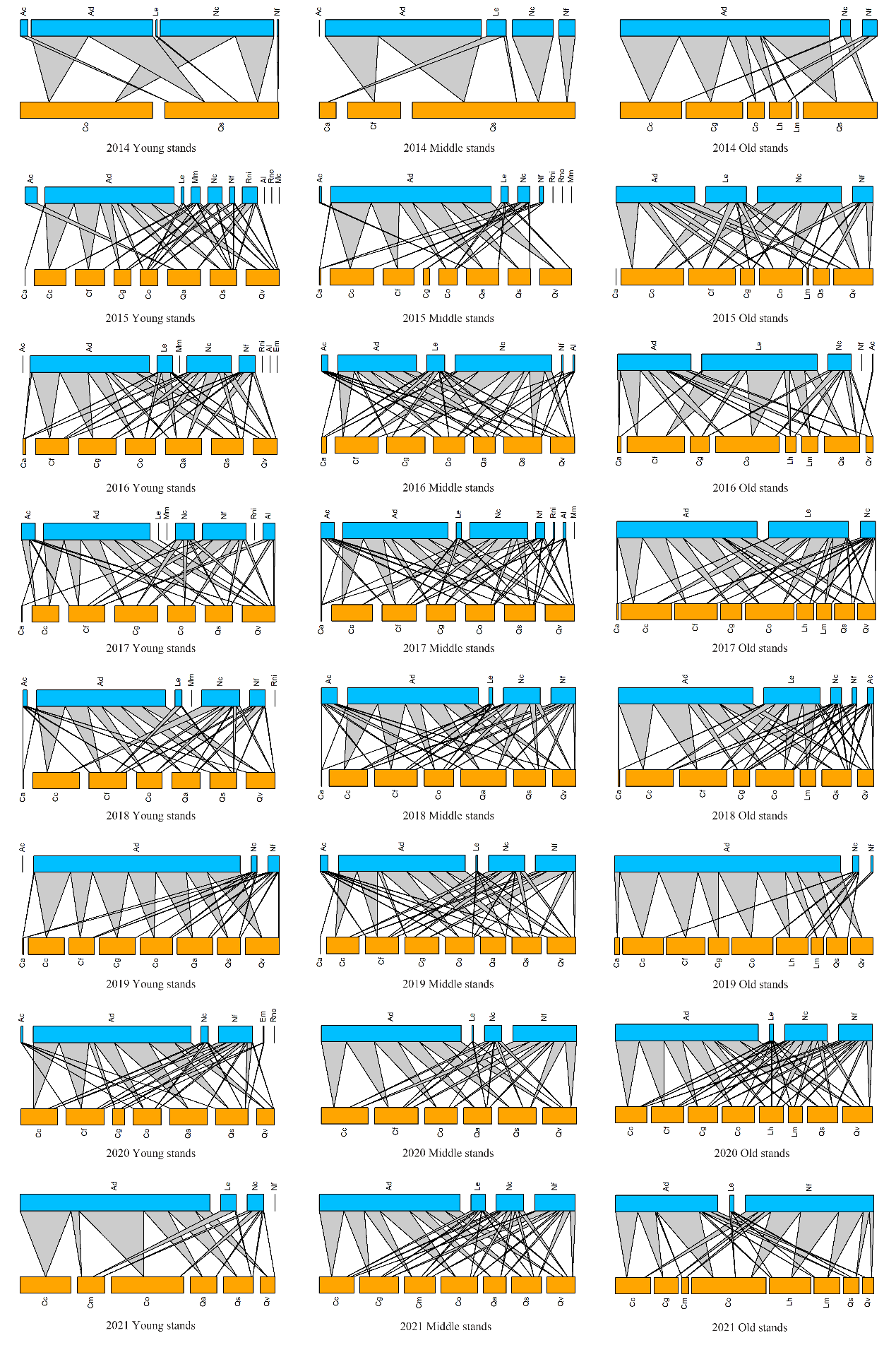


**Figure S5** Bipartite graphs of seed-rodent interaction networks in three stands over 8 consecutive years from 2014 to 2021. The size of the blue square (top) and orange square (bottom) indicated the relative abundance of the rodent and seed interaction, respectively. The grey line indicated the interaction between rodents and seeds, and the line thickness indicated the interaction strength. Rodents: Ad-*Apodemus draco*, Ac-*A. chevrieri*, Al-*A. latronum*, Le-*Leopoldamys edwardsi*, Nf-*Niviventer fulvescens*, Nc-*N. confucianus*, Rni-*Rattus nitidus*, Rno-*R. norvegicus*, Em-*Eothenomys melanogaster*, Mm-*Micromys minutus*, and Mc-*Mus musculus*, respectively. Seeds: Qa*-Quercus acutissima*, Qs*-Q. serrata*, Qv*-Q. variabilis*, Cg- *Q. glauca*, Lm*-Lithocarpus harlandii*, Lh-*L. hancei*, Ca*- Choerospondias axillaris*, Cf*-Castanopssi fargesii*, Cc*-C. ceratacantha*, Cm-*Castanea mollissima*, and Co*-Camellia oleifera*, respectively. In the figure, the left showed young stands, the middle showed middle stands, and the right showed old stands.


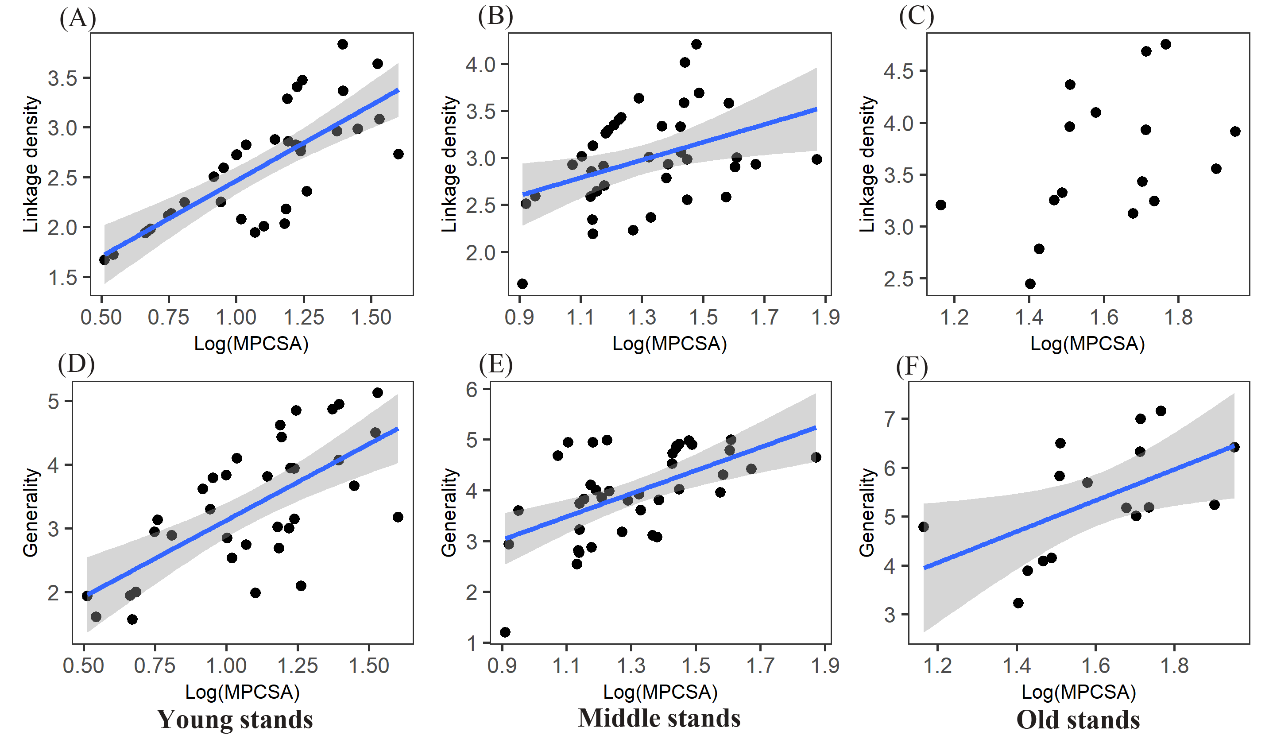


**Figure S6** Relationships between MPCSA and linkage density (A‒C) and generality (D‒F) in different stands. The left showed young stands, the middle showed middle stands, and the right showed old stands**.** MPCSA, metabolic per capita seed availability. Regression lines (with 95% confidence bands) indicate there is a significant relationship between the two variables (*P* < 0.05) based on general linear models (Table S11).


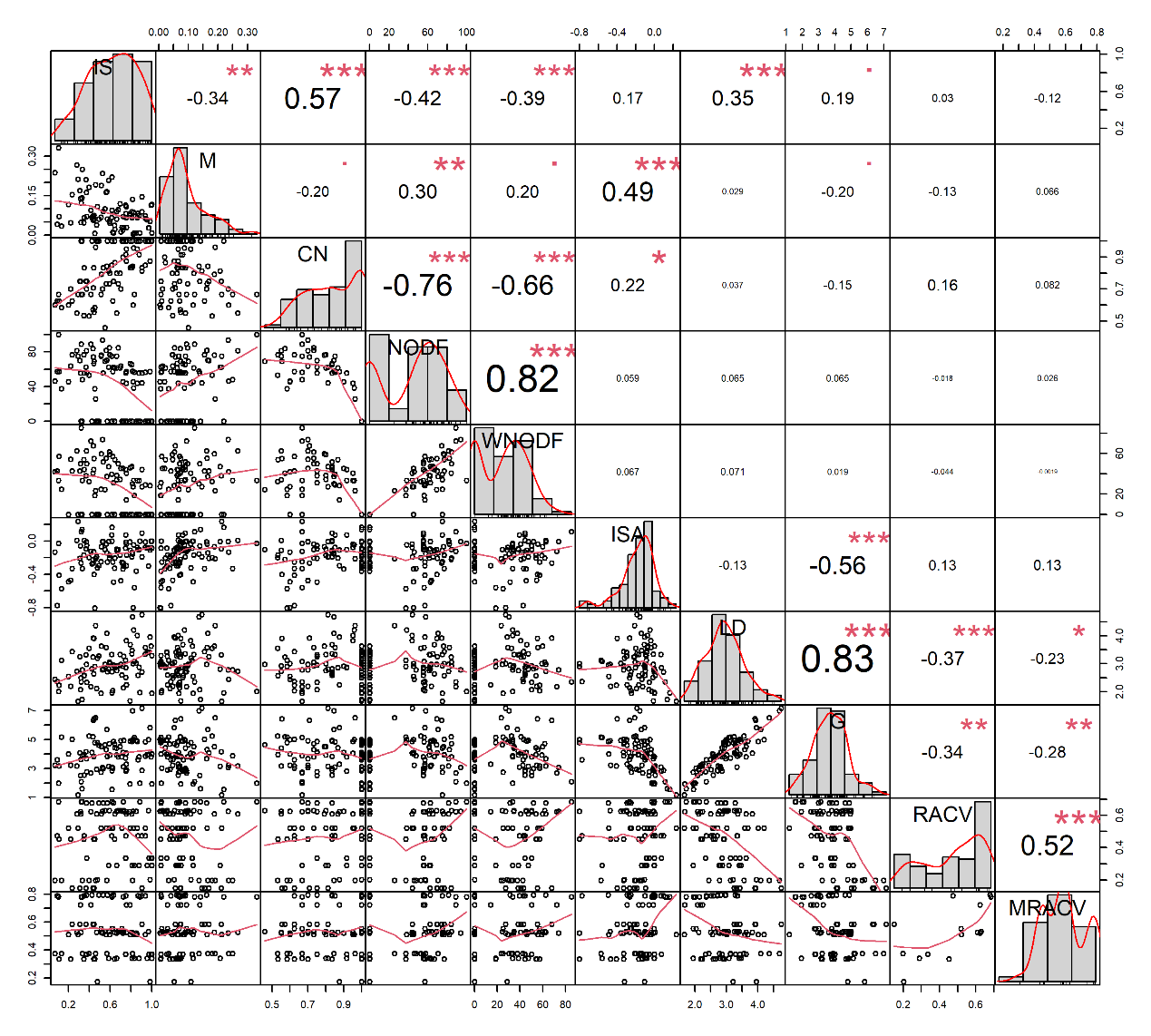


**Figure S7** Spearman correlation analysis of network metrics of seed-rodent interaction networks and rodent community stability. IS, Interaction strength; M, modularity; CN, connectance; NODF, nestedness; WNODF, weighted NODF; ISA, interaction strength asymmetry; LD, linkage density; G, generality; RACV, the CV of rodent abundance; MRACV, the CV of rodent biomass (MRA). Temporal variability of community was used to assess community stability, and temporal variability of community is commonly measured as the coefficient of variation (CV, here calculated as standard deviation/mean ×100%) (Thebault and Loreau 2005).

**References**

Dormann, C.F., Gruber, B., and Frund, J. (2008). Introducing the bipartite package: Analysing ecological networks. *R News*, 8**,** 8-11.

Menzel, A., Sparks, T.H., Estrella, N., Koch, E., Aasa, A., Ahas, R., Alm-K bler, K., Bissolli, P., Braslavsk , O.G., Briede, A., Chmielewski, F.M., Crepinsek, Z., Curnel, Y., Dahl, , Defila, C., Donnelly, A., Filella, Y., Jatczak, K., M ge, F., Mestre, A., Nordli, , Pe uelas, J., Pirinen, P., Remišov , V., Scheifinger, H., Striz, M., Susnik, A., Van Vliet, A.J.H., Wielgolaski, F.-E., Zach, S., and Zust, A.N.A. (2006). European phenological response to climate change matches the warming pattern. *Global Change Biol.*, 12**,** 1969-1976. doi:10.1111/j.1365-2486.2006.01193.x

Thebault, E., and Loreau, M. (2005). Trophic interactions and the relationship between species diversity and ecosystem stability. *The American Naturalist*, 166**,** E95-E114. doi:10.1086/444403

Yang, X., Yan, C., Zhao, Q., Holyoak, M., Fortuna, M.A., Bascompte, J., Jansen, P.A., and Zhang, Z. (2018). Ecological succession drives the structural change of seed-rodent interaction networks in fragmented forests. *For. Ecol. Manage.*, 419-420**,** 42-50. doi:10.1016/j.foreco.2018.03.023

Yodzis, P. (1981). The stability of real ecosystems. *Nature*, **289**, 674-676. Doi: 10.1038/289674a0
